# Genomic profiling of fungal cell wall-interfering compounds: identification of a common gene signature

**DOI:** 10.1186/s12864-015-1879-4

**Published:** 2015-09-05

**Authors:** Raúl García, Javier Botet, José Manuel Rodríguez-Peña, Clara Bermejo, Juan Carlos Ribas, José Luis Revuelta, César Nombela, Javier Arroyo

**Affiliations:** Departamento de Microbiología II, Facultad de Farmacia, Universidad Complutense de Madrid, IRYCIS, 28040 Madrid, Spain; Departamento de Microbiología y Genética, Universidad de Salamanca, Campus Miguel de Unamuno, 37007 Salamanca, Spain; Instituto de Biología Funcional y Genómica, Consejo Superior de Investigaciones Científicas (CSIC) / Universidad de Salamanca, 37007 Salamanca, Spain

**Keywords:** Fungal cell wall, Cell integrity, Genomics, Screening, β-1,3 glucan, Chitin

## Abstract

**Background:**

The fungal cell wall forms a compact network whose integrity is essential for cell morphology and viability. Thus, fungal cells have evolved mechanisms to elicit adequate adaptive responses when cell wall integrity (CWI) is compromised. Functional genomic approaches provide a unique opportunity to globally characterize these adaptive mechanisms. To provide a global perspective on these CWI regulatory mechanisms, we developed chemical-genomic profiling of haploid mutant budding yeast cells to systematically identify in parallel those genes required to cope with stresses interfering the cell wall by different modes of action: β-1,3 glucanase and chitinase activities (zymolyase), inhibition of β-1,3 glucan synthase (caspofungin) and binding to chitin (Congo red).

**Results:**

Measurement of the relative fitness of the whole collection of 4786 haploid budding yeast knock-out mutants identified 222 mutants hypersensitive to caspofungin, 154 mutants hypersensitive to zymolyase, and 446 mutants hypersensitive to Congo red. Functional profiling uncovered both common and specific requirements to cope with different cell wall damages. We identified a cluster of 43 genes highly important for the integrity of the cell wall as the common “signature of cell wall maintenance (CWM)”. This cluster was enriched in genes related to vesicular trafficking and transport, cell wall remodeling and morphogenesis, transcription and chromatin remodeling, signal transduction and RNA metabolism. Although the CWI pathway is the main MAPK pathway regulating cell wall integrity, the collaboration with other signal transduction pathways like the HOG pathway and the invasive growth pathway is also required to cope with the cell wall damage depending on the nature of the stress. Finally, 25 mutant strains showed enhanced caspofungin resistance, including 13 that had not been previously identified. Only three of them, *wsc1*Δ, *elo2*Δ and *elo3*Δ, showed a significant decrease in β-1,3-glucan synthase activity.

**Conclusions:**

This work provides a global perspective about the mechanisms involved in cell wall stress adaptive responses and the cellular functions required for cell wall integrity. The results may be useful to uncover new potential antifungal targets and develop efficient antifungal strategies by combination of two drugs, one targeting the cell wall and the other interfering with the adaptive mechanisms.

**Electronic supplementary material:**

The online version of this article (doi:10.1186/s12864-015-1879-4) contains supplementary material, which is available to authorized users.

## Background

The main physical barrier to protect fungal cells from osmotic shock and other challenging environmental conditions is constituted by the cell wall. The cell wall of the budding yeast *Saccharomyces cerevisiae* is formed by an inner layer containing β-1,3 glucan, β-1,6 glucan and chitin, and an outer layer composed of mannoproteins [1]. All these components are linked to each other to form a compact network whose integrity is essential for cell morphology and viability [2, 3]. Since the cell wall is essential for fungal survival and its composition is unique, this structure is an excellent target for antifungal drugs [4, 5]. In fact, several agents interfering with the synthesis of β-1,3 glucan and chitin have been developed. A good example is the echinocandin family of β-1,3 glucan synthase (GS) non-competitive inhibitors which includes micafungin, anidulafungin and caspofungin (CAS) [5, 6].

To ensure their integrity, fungal cells have evolved mechanisms to detect cell wall damage and elicit adequate adaptive responses. The major signaling pathway regulating these mechanisms is the Slt2/Mpk1 mitogen-activated protein kinase (MAPK) cell wall integrity (CWI) pathway, which is activated by conditions that compromise cell wall integrity such as chemical cell wall-perturbing agents or mutations that impair cell wall stability [7–9]. The final consequence of the activation of this pathway is the induction of an adaptive transcriptional program extensively studied by genome-wide expression profiling, that ultimately causes the remodeling of cell wall architecture for survival [10–14]. This adaptive compensatory response, which is also triggered by echinocandin-derivate antifungals, creates a new potential concept in antifungal drug treatment based on the possibility of combinatorial therapy using two drugs together, one targeting the cell wall and the other targeting the molecular mechanism of fungal adaptive response [15, 16].

Functional genomic approaches using the model organism *S. cerevisiae* provide a unique opportunity to globally study these adaptive mechanisms through the characterization of mutant collections [17], genome-wide gene expression profiling [18] and genetic interactions [19]. Genome-wide transcriptional profiles to different types of cell wall stress have shown the existence of a common transcriptional adaptive response but also the existence of specific gene expression effects for each situation [20]. Although the CWI pathway is crucial in the regulation of yeast adaptive responses against cell wall damage, other pathways are also involved. Thus, whereas transcriptional adaptive responses to the presence of Congo red (CR) almost completely depends on the transcriptional factor Rlm1 and the MAPK Slt2 [13], activation of CWI signaling in response to treatment with zymolyase (ZYM), an enzymatic cocktail containing β-1,3 glucanase, protease, mannanase and chitinase activities, requires a sequential activation by this stress of the two CWI and high osmolarity glycerol (HOG) pathways [14, 21]. Additionally, β-1,3 glucan inhibitors induce transcriptional responses only partially dependent on Rlm1 (García, R., Bravo, E., Rodriguez-Peña, JM and Arroyo, J, unpublished results), suggesting the participation of additional signaling pathways. Genome-wide surveys of genes regulated through the CWI pathway are also consistent with the co-activation of CWI signaling and Ca^2+^ signaling (Calcineurin/Crz1 pathway) as well as with general stress signaling [12, 13, 22].

Chemical-genomic profiling of bioactive compounds has been shown as a powerful approach for drug target identification and mode of action studies [23, 24]. Previous published results have addressed the identification of genes involved in cell wall biogenesis by means of individually screening mutant collections for sensitivity to calcofluor white [25, 26], K1 killer toxin [27] and CAS [28, 29]. More recently, a genome-wide survey of yeast mutations leading to the activation of the CWI pathway has been conducted [9]. Here, to gain further insight into CWI regulatory mechanisms and provide a global perspective on the mechanisms involved in the cellular adaptive responses to cell wall damage, we screened in parallel the budding yeast collection of haploid mutant strains to quantitatively measure the relative fitness of each mutant to treatments with three different cell wall perturbing agents: CR, ZYM and CAS. Our aim was to systematically identify the genes required to cope with stresses interfering the cell wall by different modes of action, thus leading to well-characterized cell wall adaptive transcriptional responses. This approach has allowed us to obtain a comprehensive view of the gene network related to cell wall homeostasis. Moreover, the comparative analysis of the genes required to tolerate the three agents under study along with those from other conditions publicly available that potentially affect fungal cell integrity, has allowed us to define a cell wall maintenance (CWM) gene signature which includes those genes necessary to resist the majority of situations in which cell wall is damaged. In addition, the characterization of the set of mutants resistant to CAS and CR revealed the significance of membrane lipids for the activity of these compounds. Therefore, this work provides a global perspective on the mechanisms involved in cell stress adaptive responses and the cellular functions required for cell wall integrity, which may be useful to uncover new potential antifungal targets.

## Results and discussion

### Chemical-genomic profiling of Congo red, zymolyase and caspofungin

To comprehensively characterize the cellular impact of the cell wall interfering compounds CR, ZYM, and CAS, we screened the budding yeast collection of 4786 non-essential haploid deletion mutants. These compounds were selected because they act on the cell wall by different stress mechanisms. CR binds to chitin interfering with proper cell wall assembly [30, 31], whereas ZYM affects cell wall integrity due to a main β-1,3 glucanase activity and noticeable protease, mannanase and chitinase activities [32–34]. Finally, echinocandins, including CAS, interfere with the β-1,3 glucan network by specifically inhibiting β-1,3 GS [35]. In agreement with their different modes of action, the transcriptional adaptive response to the three cell wall insults includes common and specific transcripts and depends on different cell surface sensors [36].

We first set up the optimal growth parameters for each condition to identify hypersensitive yeast deletion mutants relative to the wild-type strain. To obtain quantitative data about the growth levels, the screenings with ZYM and CAS were performed in rich liquid medium (YEPD) using a microtiter plate format and monitoring the growth of each strain by measuring the absorbance at 595 nm at different time points. In the case of Congo red, we used a spot dilution assay on YEPD solid media.

Key parameters to be defined were the optimal drug concentration and the growth conditions to be used. Pilot assays, in which we measured the growth of the wild type strain in the presence of different concentrations of each compound, were designed to determine the higher drug concentration that did not significantly affect the growth of the wild type strain, named as sub-MIC (minimal inhibitory concentration) (Additional file 1). Additionally, some reference strains related to cell wall stress response and known to be hypersensitive to the compounds were also included, like the mutant in the MAPK of the CWI pathway (*slt2*Δ) [13, 14, 28]. On the basis of these results, we finally selected sub-MIC doses of 30 U/ml ZYM and 50 μg/ml CR. In the case of CAS, the range of hypersensitivity between the wild type and *slt2*Δ strains was narrower (Additional file 1). Therefore, we decided to include three sub-MIC doses (10 ng/ml, 20 ng/ml and 30 ng/ml) in this screening. All these concentrations do not affect the growth of the wild-type strain but clearly inhibit the growth of the reference hypersensitive strains.

**Fig. 1:**
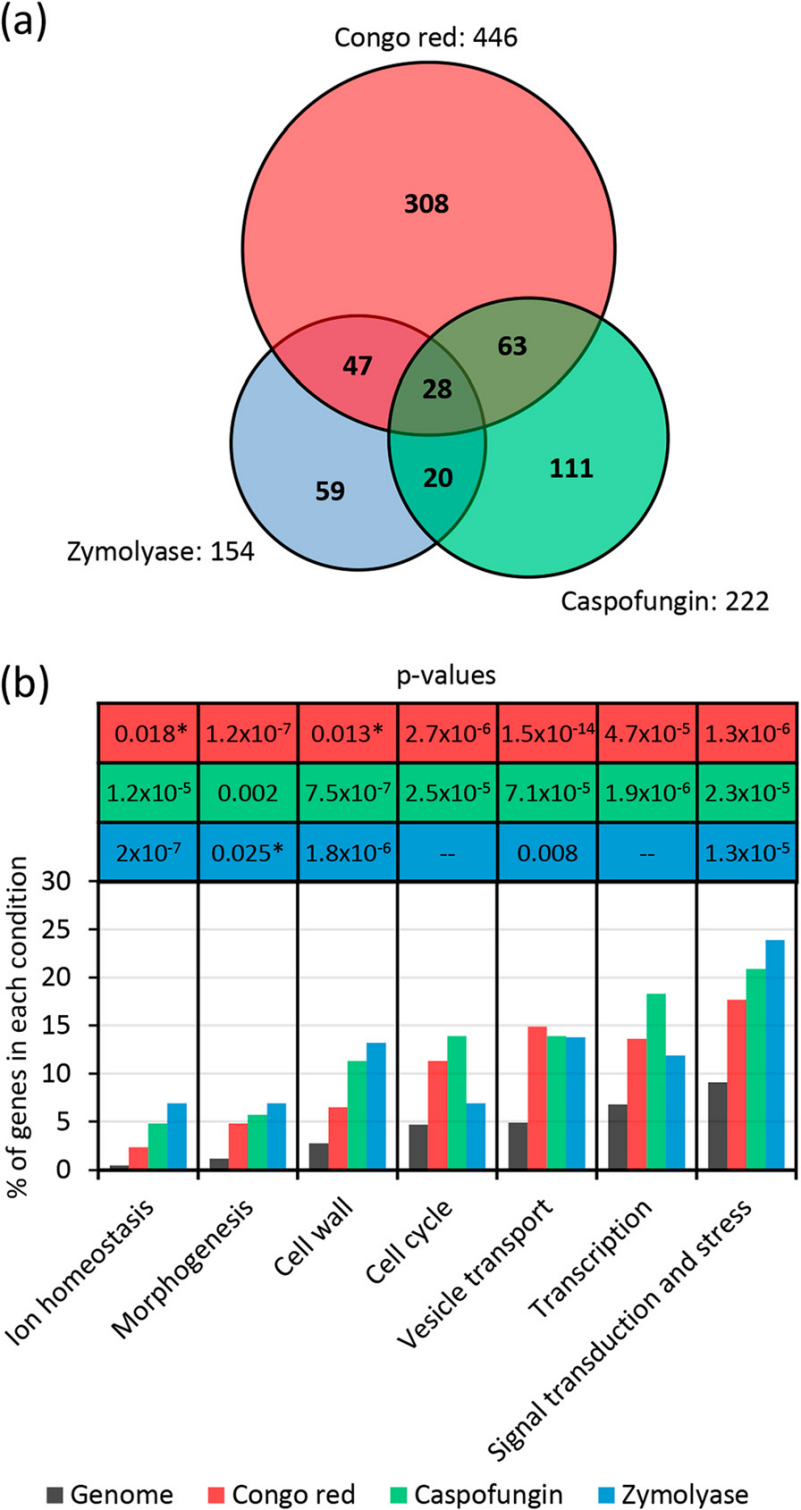
Global analysis of hypersensitive phenotypes to Congo red, caspofungin and zymolyase. **a** Venn diagram summarizing the distribution of yeast deletion mutants hypersensitive to the cell wall interfering compounds. **b** Functional classification of genes identified in the three genome-wide screenings according to the Gene Ontology term finder tool (*p*-value <0.01). Only the statistically significant functional groups identified are shown. *p*-values between 0.010-0.025 are labeled with an asterisk

Next, we quantitatively measured, for each compound-dose, the relative fitness of the whole collection of 4786 haploid yeast knock-out mutants. After removing those mutants that showed reduced growth in YEPD medium in the absence of stress (slow-growth phenotype) to avoid misinterpretation of the hypersensitivity levels, we proceeded to confirm the positive hits in a second round of phenotypic analysis. For CAS, the original screening protocol was repeated confirming that all mutant strains formerly detected showed hypersensitivity except five of them. For secondary screening of zymolyase hypersensitive strains, we determined their MICs to zymolyase (see Methods for details), confirming about 90 % of positive hits. In the case of Congo red because the high number of mutants selected (446 strains), we retested 13 % of them (58 strains), corroborating the sensitivity to CR in about 93 % of the strains. Eventually, we identified 222 mutants sensitive to CAS, 154 mutants sensitive to ZYM, and 446 mutants sensitive to CR (Fig. 1a). Mutants were arbitrarily ascribed to three categories (1–3) based on their sensitivity level (low, medium and high, respectively) as described in Methods. The complete dataset of the 636 yeast mutants identified in our genomic profiles including information about their degree of growth alteration is described in Additional file 2. Two previous genome-wide phenotypic analyses using CAS have been published [28, 29]. However, probably due to differences in the experimental methodology, drug batch and drug concentrations used, we have identified a significantly higher number of CAS sensitive strains. Lesage and colleagues identified 52 CAS-sensitive strains, of which 34 (68 %) are common with our CAS-sensitive gene set, whereas Markovich *et al.* identified 20 sensitive strains, of which 17 (90 %) are coincident with our set.

To determine those enriched functional categories represented within the 636 candidate hits, we used the Gene Ontology (GO) tool “GO Term Finder”. A significant enrichment for GO biological process terms related to cell wall biogenesis, morphogenesis, stress and signal transduction, but also to vesicle transport, transcription, ion homeostasis, and cell cycle (Fig. 1b) was found, suggesting that all these cellular processes are likely to be important to cope with cell wall stress. For a complete functional cataloging of the identified mutants, we used the GO tool “GO Slim Mapper”, further refining the search with annotations of the BIOBASE Knowledge Library Proteome and the *Saccharomyces* Genome Database (SGD) (Additional file 2).

### Genome-wide functional profiling uncovers common and specific requirements to cope with different cell wall damages

Comparing the mutant strains identified in the three screenings, a limited number of strains displayed overlaping fitness profiles with at least two compounds, including 75, 91, and 48 mutants common to CR-ZYM, CR-CAS, and ZYM-CAS treatments, respectively (Fig. 1a). Moreover, a core of 28 strains were hypersensitive to treatment with the three compounds tested (Table 1). The set of genes within this group must be essential to withstand cell wall damages of different origin and includes essential functions required to tolerate cell wall stress independently of the nature of the damage. This core of genes, further described below, is enriched in general functions related to vesicular trafficking and transport (mainly related to V-ATPase activity), cell wall and morphogenesis, transcription and chromatin remodelling, and signal transduction (Table 1). This group also contains some genes that function in multidrug resistance (MDR) response [37–39]. Despite the fact that there was a low number of overlaping genes affecting cell fitness in the three conditions under study (28 out of 636 detected in at least one condition), the proportion of mutant strains affected in specific cellular functions was similar for each chemical-genomic profile (Fig. 1b). Thus, although several molecular processes are common to CR, CAS and ZYM adaptive responses, specific gene/proteins are required to cope with distinct cell wall alterations, showing the complexity and versatility of the cellular cell wall stress response network. An aspect to remark is the high number of strains (308) that only showed a growth defect in the presence of CR, in contrast to the effect observed for CAS (111) and ZYM (59), suggesting that CR exerts a broader cell wall injury.

**Table 1:** Mutant strains hypersensitive to Congo red, caspofungin, and zymolyase

ORF	Gene	Functional group	Description
*YML008C* ^a^	*ERG6*	carbohydrate and lipid metabolism	Methyltransferase, converts zymosterol to fecosterol in the ergosterol biosynthetic pathway
*YBL058W* ^a^	*SHP1*	cell cycle	UBX domain-containing protein, promotes cell cycle progression by positive regulation of Glc7
*YAL023C*	*PMT2*	cell wall and morphogenesis	Protein O-mannosyltransferase of the ER membrane
*YDR245W*	*MNN10*	cell wall and morphogenesis	Subunit of a Golgi mannosyltransferase complex
*YNL084C* ^a^	*END3*	cell wall and morphogenesis	EH domain-containing protein involved in endocytosis and cell wall and cytoskeletal organization
*YOR035C* ^a^	*SHE4*	cell wall and morphogenesis	Protein containing a UCS domain, binds to myosin motor domains to regulate myosin function
*YGL173C*	*KEM1*	protein and RNA metabolism	5'-3' exonuclease, component of cytoplasmic processing (P) bodies involved in mRNA decay
*YKL054C* ^a^	*DEF1*	protein and RNA metabolism	RNAPII degradation factor, enables ubiquitination of RNAPII in an elongation complex
*YJL080C* ^a^	*SCP160*	protein and RNA metabolism	Essential RNA-binding G protein effector of mating response pathway
*YDL006W* ^a^	*PTC1*	signaling pathways and response to stress	2C protein phosphatase involved in the dephosphorylation of multiple MAP kinases
*YDR264C*	*AKR1*	signaling pathways and response to stress	Palmitoyl transferase, acts as a negative regulator of pheromone response pathway
*YDR477W* ^a^	*SNF1*	signaling pathways and response to stress	AMP-activated serine/threonine kinase involved in derepression of glucose-repressed genes
*YHR030C* ^a^	*SLT2*	signaling pathways and response to stress	Serine/threonine MAP kinase of the CWI pathway involved in maintenance of cell wall integrity
*YJL095W* ^a^	*BCK1*	signaling pathways and response to stress	MAPKKK acting in CWI signaling pathway
*YCR020W-B*	*HTL1*	transcription and chromatin remodeling	Component of the RSC chromatin remodeling complex
*YBL093C*	*ROX3*	transcription and chromatin remodeling	Subunit of the RNA polymerase II mediator complex
*YJL115W*	*ASF1*	transcription and chromatin remodeling	Nucleosome assembly factor involved in chromatin assembly and disassembly
*YOL148C* ^a^	*SPT20*	transcription and chromatin remodeling	Subunit of the SAGA transcriptional regulatory complex
*YEL044W* ^a^	*IES6*	transcription and chromatin remodeling	Component of the INO80 chromatin remodeling complex
*YJL204C* ^a^	*RCY1*	vesicular trafficking and transport	F-box protein involved in recycling endocytosed proteins
*YLL043W*	*FPS1*	vesicular trafficking and transport	Aquaglyceroporin, plasma membrane channel, involved in efflux of glycerol and xylitol
*YLR138W*	*NHA1*	vesicular trafficking and transport	Na+/H+ antiporter involved in sodium and potassium efflux through the plasma membrane
*YOR332W* ^a^	*VMA4*	vesicular trafficking and transport	Subunit E of the V1 domain of the vacuolar H + −ATPase (V-ATPase)
*YKL080W* ^a^	*VMA5*	vesicular trafficking and transport	Subunit C of the V1 peripheral membrane domain of V-ATPase
*YGR020C* ^a^	*VMA7*	vesicular trafficking and transport	Subunit F of the V1 peripheral membrane domain of V-ATPase
*YGR105W*	*VMA21*	vesicular trafficking and transport	Integral membrane protein required for V-ATPase function
*YHR060W* ^a^	*VMA22*	vesicular trafficking and transport	Protein that is required for vacuolar H + −ATPase (V-ATPase) function
*YKL119C* ^a^	*VPH2*	vesicular trafficking and transport	Integral membrane protein required for V-ATPase function

Those gene deletions that have been associated to multidrug resistance (MDR) phenotype are labeled with superscripted “a”

#### Cell wall and morphogenesis related genes

Taken together, cell wall and morphogenesis categories included about 30 % of the hypersensitive mutants identified in our chemical-genomic screenings. For CAS, in addition to the prototypical cell wall hypersensitive *fks1*Δ mutant (*FKS1* encodes a catalytic subunit of β-1,3 GS), mutants deleted in genes involved in chitin synthesis such as *CHS3* and *SKT5* (directly related to chitin synthase III activity), *CHS5*, *CHS6* and *CHS7* (involved in Chs3 export) and *BNI4* (required for chitin synthase III localization) were also identified. The level of cell growth inhibition induced by CAS of these chitin-related mutants was very similar, reinforcing the idea that the chitin generated by CSIII (chitin synthase III activity) becomes essential to strengthen the cell wall when the β-1,3-glucan synthesis is blocked.

A subgroup of mutants in genes related to cell wall glucan metabolism, including *fks1*Δ and *kre6*Δ (β-1,3 and β-1,6-glucan synthesis), *cwh41*Δ (involved in β-1,6-glucan assembly), *gas1*Δ and *gas5*Δ (β-1,3 glucanosyltransferases involved in β-1,3 glucan crosslinking), *crh1*Δ (chitin-glucan crosslinking) and *yps7*Δ and *mkc7*Δ (GPI-anchored aspartyl proteases necessary for cell wall maintenance), showed reduced fitness to CR treatment. Additionally strains lacking *FKS1* and *CWH41,* or *KRE6* and *GAS1* were also hypersensitive to CAS or ZYM, respectively, clearly indicating that β-1,3 glucan or a more general glucan integrity network are essential to circumvent their effects. In contrast, deletion of *FKS2*, which encodes for the alternative β-1,3 GS catalytic subunit, was not identified in any of the screenings, in spite of being transcriptionally induced under cell wall stress conditions [13].

Genes necessary for the mannosylation of cell wall proteins (*PMT2* and *MNN10*), endocytosis and organization/polarization of the actin cytoskeleton (*END3 and SHE4*) were required to tolerate the damage generated by the three compounds (Table 1). Highlighting the importance of cell wall protein mannosylation for the integrity of the cell wall, Mnn11, Ktr6, Kre2, Mnn2, and Hoc1 mannosyltransferases were necessary for CR tolerance, while Och1, Cwh8 (both also necessary to grow in presence of CAS), Mnn9, Pmt1, Ecm33, Cwh36 and the aforementioned Hoc1 and Mnn11 were required for growing in the presence of ZYM. These results are in agreement with previous observations showing different levels of hierarchy in maintaining cell wall homeostasis for mannosyltransferases, despite being highly redundant genes [9, 40, 41].

Interestingly, we found that mutants associated to microtubule motor proteins and/or cytoskeletal organization (*CIN8*, *KAR3*, *NIP100*, *KIP1*, *CIK1*, *PAC11, TPM1, NUM1* or *JNM1*) were specifically sensitive to CR. This observation points to a link between cell wall alterations caused by CR and microtubule associated functions, although some of these mutations could also confer pleiotropic phenotypes.

Altogether, these results further support the existence of specific compensatory mechanisms, affecting chitin and glucan structure, when cell wall integrity is jeopardized and reinforce the functional relationship between cell wall maintenance and morphogenesis. Thus, genes related to bud emergence, actin cortical patch assembly and, mainly bud site selection were also broadly identified (Additional file 2).

#### MAPK signal transduction pathways and response to stress

**Fig. 2:**
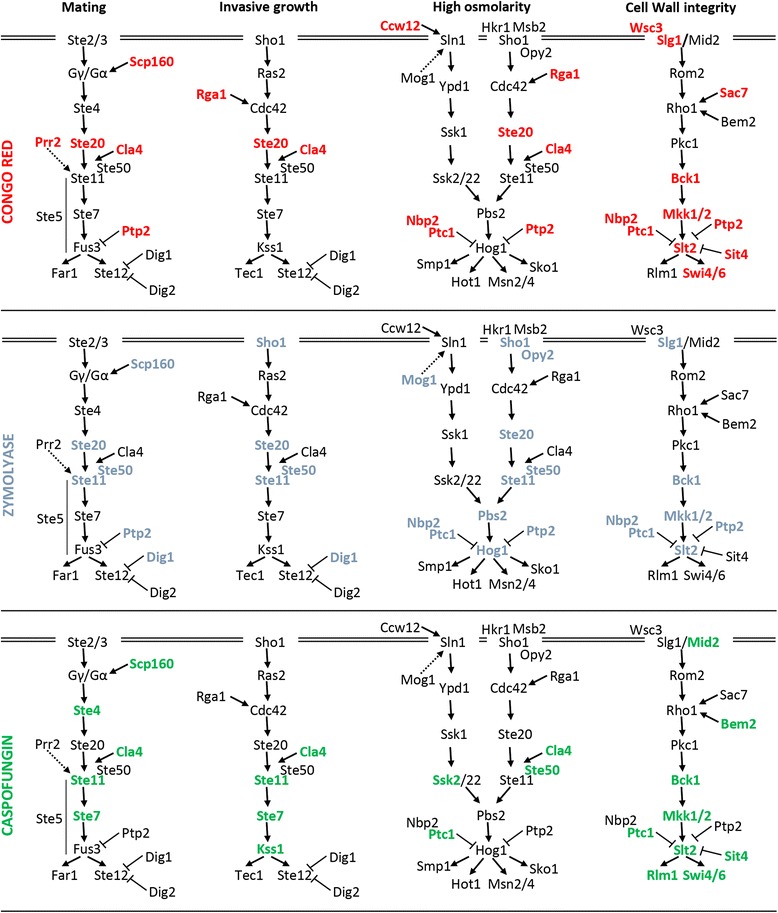
Yeast MAPK signaling pathways are associated to hypersensitivity to cell wall stress conditions. MAPK pathway elements whose deletion causes hypersensitivity to CR, ZYM or CAS are labeled in *red*, *blue* or *green*, respectively

During the last decade, a great amount of information has been accumulated about the participation of MAPK pathways, particularly the CWI pathway, in the regulation of adaptive responses to cell wall damage [7, 20]. Our results suggest that, although the CWI pathway is the main pathway regulating cell wall integrity, cooperation with other signal transduction pathways is also required. The architecture of the different yeast MAPK pathways indicating those elements identified in our genomic profiles is shown in Fig. 2. In agreement with an essential role of the CWI pathway, deletion of key elements of this pathway like the MAPKKK Bck1 and the MAPK Slt2 induced hypersensitivity to the three compounds (CR, CAS and ZYM). Combined deletion of the two redundant MAPKKs of this pathway (*MKK1* and *MKK2*) is necessary to cause sensitivity to CR and ZYM [13, 14]. However, the single mutants were sensitive to CAS, supporting the hypothesis that these two proteins could play some non-redundant functions [42]. Regarding the sensors of the CWI pathway, deletion of *WSC1/SLG1* and *WCS3* caused sensitivity to CR, while *MID2* deletion caused CAS sensitivity. Paradoxically, damage caused by CR is mainly sensed by Mid2, while Wsc1 senses CAS damage [36, 43]. These results might be explained in the light of the blockade of the glucan-chitin compensatory system, since Wsc1 directly regulates the GS Fks1 [44], while Mid2 regulates chitin synthesis under stress conditions [45]. In addition, Sac7 and Bem2, two Rho-type GTPase-activating proteins (GAPs), are necessary to address the damage caused by CR, and CAS, respectively, supporting the idea that different Rho1 regulators elicit specific outputs.

It is well known that fine regulation of MAPK pathways is essential in eukaryotic physiology. In this context, several mutants in genes encoding protein phosphatases associated to MAPK signaling pathways displayed altered growth fitness to the compounds tested. This was the case of *PTP2* (acts on Hog1, Fus3 and Slt2) required to growth in the presence of CR and ZYM, *SIT4* (negative regulator of Pkc1) whose absence renders cells hypersensitive to CR and CAS, and *PPS1* (which codes for a dual specificity protein phosphatase not previously linked to MAPK activity), required for CR tolerance. Moreover, the growth of the *ptc1*Δ mutant strain in presence of all three compounds was drastically inhibited, denoting the relevance of this phosphatase under cell wall stress conditions. In agreement, the *ptc1*Δ *slt2*Δ double mutation is synthetically lethal [46] and mutations of *PTC1* results in the increased expression of genes that are also induced by cell wall damage [47].

In addition to elements of the CWI pathway, all the components of the Sho1 branch of the high-osmolarity glycerol (HOG) pathway (Sho1, Opy2, Ste20, Ste50, Ste11, Hog1 and Pbs2) were required to hinder the damage caused by ZYM (Fig. 2 and Additional file 2) in agreement with our previously reported cooperation between the HOG and CWI pathways to respond against cell wall stress caused by ZYM [14, 21]. Furthermore, the absence of *DIG1* (MAP kinase-responsive inhibitor of the Ste12 transcription factor, involved in the regulation of mating-specific genes and the invasive growth pathway) and *RHO4* (a small GTPase likely involved in the establishment of cell polarity) also caused sensitivity to ZYM, which might be indicative of their participation in the dual regulation by CWI and HOG pathways. How the signal from the HOG pathway is transmitted to the CWI route is still unknown, thus future characterization of these mutants could be useful to address this issue.

Interestingly, the entire MAPK module of the invasive growth pathway, *STE11, STE*7, and *KSS1,* in addition to the CWI pathway, was required for CAS tolerance (Fig. 2), pointing to a connection between CAS and invasive growth.

Deletion of *AKR1* and *SNF1* also led to strains hypersensitive to the three compounds (Table 1). *AKR1* encodes a palmitoyl transferase, being palmitoylation a reversible lipid modification that regulates membrane tethering for key proteins in cell signaling [48, 49]. Palmitoylated proteins Yck1, Yck2 and Meh1, known substrates of Akr1 were identified as hypersensitive in some of our screenings, further supporting a crucial role for the palmitoylation mediated by Akr1 for the signaling network required to cope with cell wall stress. The identification of *SNF1* as a common target of the three screenings could be related to a metabolic imbalance caused by the reduced levels of glucose-6-phosphate in the absence of this AMP-activated serine/threonine protein kinase, but it has also been recently suggested an independent role of this protein from carbohydrate signaling in cell wall integrity [50].

#### Transcription and chromatin remodeling

The sensitivity to cell wall damage of a large number of mutants deleted in genes related to transcription and chromatin remodeling processes was somehow expected because cellular adaptation to cell wall damage mainly implies transcriptional regulation. In this regard, we found genes encoding diverse elements of the transcriptional machinery such as RNA polymerase II-associated proteins or complexes (namely PAF, Mediator, Elongator and CTDK-1 protein kinase) (see Additional file 2). For the CAS screening the three subunits (*CTK1, CTK2* and *CTK3*) of the C-terminal domain kinase I (CTDK-I) were identified. The sensitivity of this set of mutants might be related to the affected transcription of genes regulated through the CWI pathway although additive effects of cell wall damage and other cellular functions that are constitutively affected in these mutants cannot be ruled out.

Moreover, we found a significant number of mutants affected in chromatin remodeling and modification, for which a critical role in establishment and maintenance of transcriptional programs under stress conditions have been established [51–53]. Chromatin structure is regulated both by chromatin remodelers and factors that covalently modify histones allowing transcription factor accessibility at RNA Pol II promoters. Within the first group, we identified mutants in elements of the SWI/SNF complex, the SWR1 complex and the RSC complex (Additional file 2). In agreement, we have recently characterized the role of the SWI/SNF complex mediating the chromatin remodeling necessary for adequate transcriptional responses to cell wall stress [53].

Regarding histone modifications, we found that several subunits of the histone acetyltransferase complex SAGA were required to tolerate the damage generated by cell wall stress, particularly by CR. In agreement, we have also recently identified several subunits of this complex in a transcriptional screening developed to identify mutants defective in the induction of *MLP1*, a transcriptional reporter of the CWI pathway [53]. This complex seems to cooperate with the SWI/SNF complex for chromatin remodeling of genes regulated through the CWI pathway under cell wall stress conditions (Sanz AB, García, R., Rodriguez-Peña, JM, Arroyo, J, unpublished results). In parallel, another 12 mutants, mainly CR and CAS hypersensitive, related to events of histone acetylation (HAT) and deacetylation (HDAC), like elements of the Hda1-associated complex (*hda1*Δ, *hda2*Δ and *hda3*Δ strains), and elements of the Compass complex, involved in histone H3 methylation were also identified. All these elements play key regulatory mechanisms for both repression and activation of gene expression.

Within this functional group, an aspect of interest was to analyze in depth a group of 16 strains deleted in different transcription factors. In theory, these factors should positively regulate the expression of genes required to grow in presence of the compounds under study. Some of them regulate global responses to particular cellular conditions such as Stp1 and Gln3, which participate in aminoacid metabolism; Rox1, repressor of hypoxic genes; Aft1, involved in iron uptake and utilization; or the general transcriptional repressor complex Ssn6-Tup1. In addition, we found altered growth fitness for mutants in elements of the Ccr4-Not complex and the SBF complex. In accordance with the observed sensitivity, loss of Ccr4 causes a decreased transcription of several genes involved in cell wall biosynthesis [54] and the heterodimeric transcriptional complex SBF, composed of Swi4 and Swi6, is required for cell-cycle-regulated transcription of cell wall biosynthetic genes and for transcriptional activation of several genes through a non-catalytic mechanism mediated by the MAPK Slt2 [55]. In fact, mutation of either *SWI4* or *SWI6* results in sensitivity to calcofluor white [56], a fluorochrome with a proposed mode of action on the cell wall similar to CR. Strikingly, loss of Rlm1, a key player in the transcriptional response to cell wall damage [10, 13], only resulted in cells sensitive to CAS, and even promoted resistance to CR (data not shown).

Five genes of this functional category were sensitive to the three compounds (Table 1): *ROX3* (encoding a subunit of the RNA polymerase II mediator complex); *ASF1* (involved chromatin assembly and disassembly); *HTL1* (a component of the RSC chromatin remodeling complex); *IES6* (component of the INO80 remodeling complex) and *SPT20* (encoding a subunit of the SAGA transcriptional regulatory complex), supporting their importance for a general cell wall adaptive response. However, although elements of the different complexes within this functional group were identified for the three compounds, a high degree of specificity in the cellular response to each compound was also found (Additional file 2), suggesting that transcriptional regulation also requires particular subunits of large protein complexes (subcomplexes), to deal with different types of stresses on the cell wall.

#### Protein and RNA metabolism

As shown in Additional file 2, our chemical genomic profiles revealed a large number of mutants in genes related to RNA metabolism, translation (ribosomal proteins), amino acid metabolism and protein modifications, in agreement with a post-translational regulation of the compensatory mechanism. It is noteworthy that 19 mutants deleted in genes involved in ubiquitination-related processes were sensitive, mainly to CR and/or CAS. Ubiquitination contributes to the regulation of numerous cellular processes, including cell cycle progression, gene transcription and stress responses [57, 58]. Thus, our data corroborate the importance of this type of modification in the regulation of responses to cell wall stress. Three genes, *KEM1, DEF1* and *SCP160*, encoding proteins involved in mRNA decay, ubiquitination of RNA PolII and translation respectively, were identified in the three screenings. The identification of Scp160 is quite interesting. This RNA-binding protein is required for efficient translation of a subset of mRNA via tRNA recycling. A recent genome-wide survey of mRNAs affected in translation as consequence of a *SCP160* deletion included an enrichment in mRNAs encoding cell wall proteins [59], although these mRNAs do not correspond to those cell wall related genes induced in the transcriptional CWI response. Our results open the possibility that Scp160 plays an essential role in translation of the pool of mRNAs over-produced under cell wall stress conditions through the CWI pathway. In this regard, it has been recently proposed that selective nuclear mRNA degradation controls the cell wall stress response [60].

The GO functional group “tRNA wobble uridine modification” was clearly enriched in strains hypersensitive to CAS and CR (*SIT4, NCS6, UBA4, URM1, KTI12, IKI3, TRM9, ELP2, ELP3, ELP4*). This is probably related to their essential role in efficient translation of cell wall stress mRNAs, as it has been recently demonstrated in *Schizosaccharomyces pombe* for oxidative stress [61].

#### Lipid metabolism

Mutants lacking genes associated to metabolism showed a significant enrichment in the category of lipid metabolism. These mutants were primarily affected in ergosterol biosynthesis, such as *erg2*Δ*, erg3*Δ*, erg4*Δ*, erg5*Δ or *erg6*Δ*,* and metabolism of sphingolipids (*arv1*Δ and *isc1*Δ). In accordance with several screens that have identified *erg6*Δ mutants with increased sensitivity to a broad range of chemical compounds, in our hands this strain was hypersensitive to the three compounds tested. It is well known that the ergosterol pathway is closely connected with sphingolipid metabolism, which has important structural and signaling functions in fungal and animal cells [62]. Moreover, *erg* mutants are defective in pheromone signaling and lipid rafts association of β-1,3 glucanosyltransferase Gas1 [63, 64]. The importance of sterols for yeast survival is evidenced by the large number of antifungal compounds affecting this route. Because the ergosterol and sphingolipid pathways constitute complex systems, it is difficult to predict which specific steps could provide most effective drug targets and what would be the consequences of the alteration of a particular enzymatic activity. However, our data uncover alternative drug targets that could be useful in combined therapies of cell wall-interfering compounds and ergosterol biosynthesis interfering drugs. Reinforcing this idea, it was recently reported that in the *arv1*Δ mutant the CWI route is constitutively activated indicating the presence of an altered cell wall [9].

#### Vesicular trafficking and transport

**Fig. 3:**
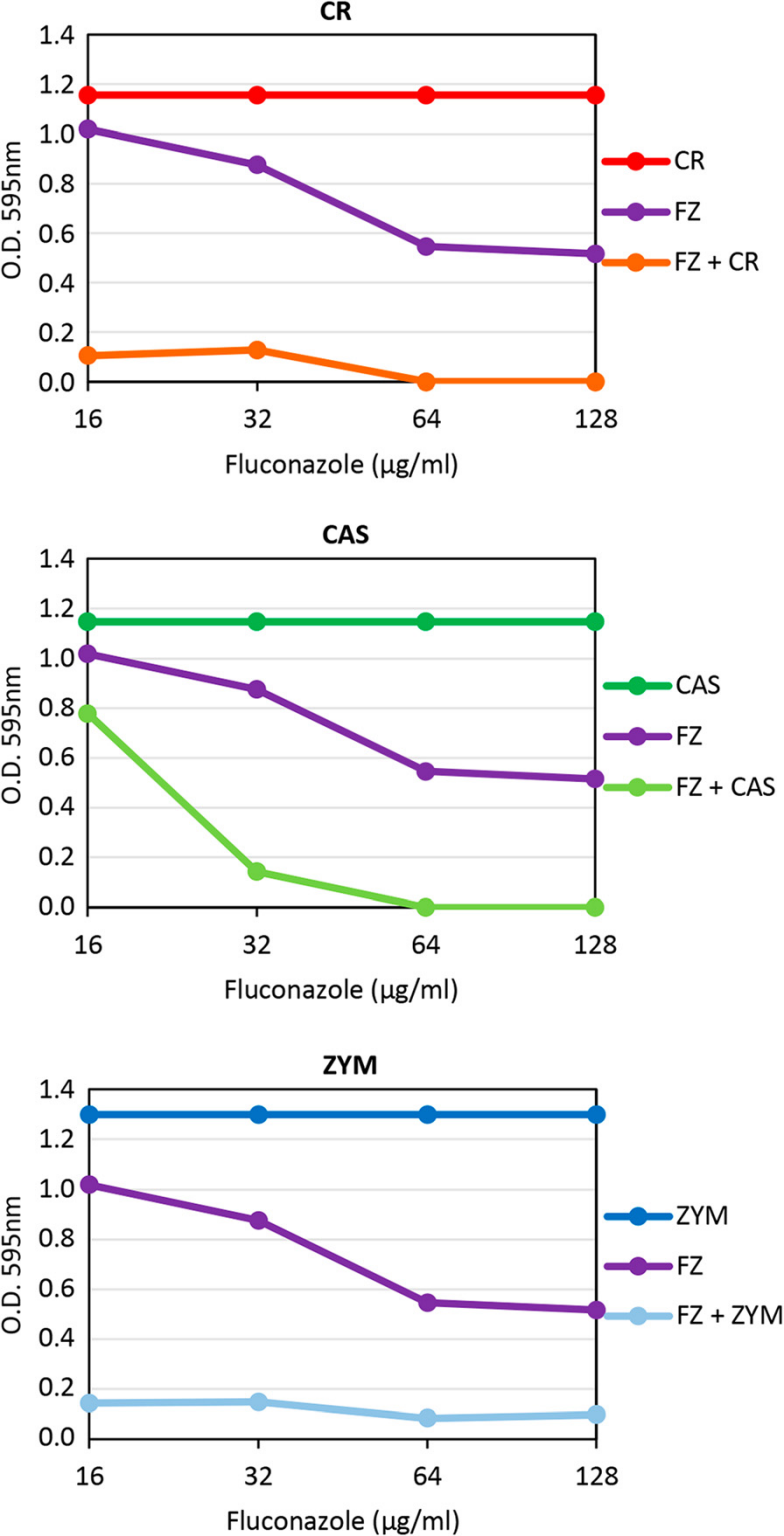
Combination of fluconazole and CR, CAS or ZYM exhibits a synergistic growth-inhibitory effect on yeast cells. Cell growth assays were carried out in 96-well microtiter plates, preparing two-fold serial dilutions of fluconazole (FZ) to give concentrations ranging from 128 to 16 μg/ml in a final volume of 150 μl of YEPD, either including a constant concentration of CR (30 μg/ml, FZ + CR), CAS (15 ng/ml, FZ + CAS) or ZYM (64 U/ml, FZ + ZYM) or not including the cell wall stress (FZ). Cultures containing only CR, CAS or ZYM were also included as growth control. Representative experiments for each condition are shown

This is one of the largest functional groups identified in our chemical genomic profiles. Encompasses a wide range of cellular trafficking-related systems such as VPS vacuolar sorting, endosomal sorting complexes (ESCRT), SNARE complex and the V-type H^+^-ATPase, among others. Six mutants in *VMA* genes (*vma4*Δ, *vma5*Δ, *vma7*Δ, *vma21*Δ, *vma22*Δ *and vph2*Δ), corresponding to different V-ATPase subunits, were highly sensitive to CR, CAS and ZYM (Table 1), demonstrating the importance of the V-ATPase activity under these conditions. The vacuolar proton-translocating ATPase (V-ATPase) is a multi-subunit complex divided in two sectors and couples energy from ATP hydrolysis (the V_1_ sector) to the transport of protons against transmembrane gradients (the Vo sector). V-ATPase also acidifies compartments of the biosynthetic pathway such as the late Golgi apparatus and secretory vesicles. The maintenance of these acidic compartments is crucial for vesicle trafficking, in both endocytic and biosynthetic pathways [65]. Indeed, it has been recently reported that loss of V-ATPase activity leads to constant low-level activation of the HOG pathway, and that the V-ATPase itself is a salt-activated enzyme in yeast, suggesting that V-ATPase acts in parallel with the HOG pathway for salt detoxification [66]. The recent description of ergosterol requirement for the V-ATPase function uncovered a critical role for ion homeostasis downstream of azole inhibition of ergosterol biosynthesis and suggested that combining azole drugs and agents affecting ion fluxes would have synergistic antifungal effect [67]. Bearing this in mind, we were prompted to test if inhibition of the V-ATPase activity by fluconazole leads to a reduction in fitness of yeast cells in the presence of cell wall stress caused by CR, CAS or ZYM. To approach this, we measured cell growth of wild-type yeast cells treated with different amounts of fluconazole ranging from 16 to 128 μg/ml either in the absence or in the presence of sublethal concentrations of CR, CAS or ZYM (30 μg/ml, 15 ng/ml and 64 U/ml, respectively). As shown in Fig. 3, simultaneous inhibition of the V-ATPase by fluconazole and cell wall interference by CR, CAS or ZYM clearly leads to a synergistic antifungal effect.

#### Genes of unknown function

One of the main objectives of a chemical-genomic profiling is to contribute to the characterization of genes with no function assigned. Mutants that show similar chemical-genomic profiles are often involved in a common or related biological process. In this regard, 66 mutant strains deleted in genes encoding proteins of unknown function or with only predicted functions assigned were unraveled. 21 of these genes were discarded because their loci overlapped in the complementary strand with other genes identified in our screening. 19 additional genes are labeled in SGD as dubious open reading frame and partially overlap with other verified genes. The remaining 26 genes (Additional file 2) potentially include new genes related to cell wall homeostasis. Included in this group are: *THP3* (probably involved in transcription elongation), *YEL043W* and *SLM6* (probably related to cytoskeleton), *NNF2* (interacts with Rpb8 RNA PolII subunit), *SYH1* (possibly involved in COPII vesicle formation) and *YLR177W*, which is a *PSP1* paralog (*psp1*Δ mutant strain was found to be hypersensitive to CR), among others.

### Correlation between chemogenomic and transcriptional profiles upon CR, CAS, and ZYM treatment

**Fig. 4:**
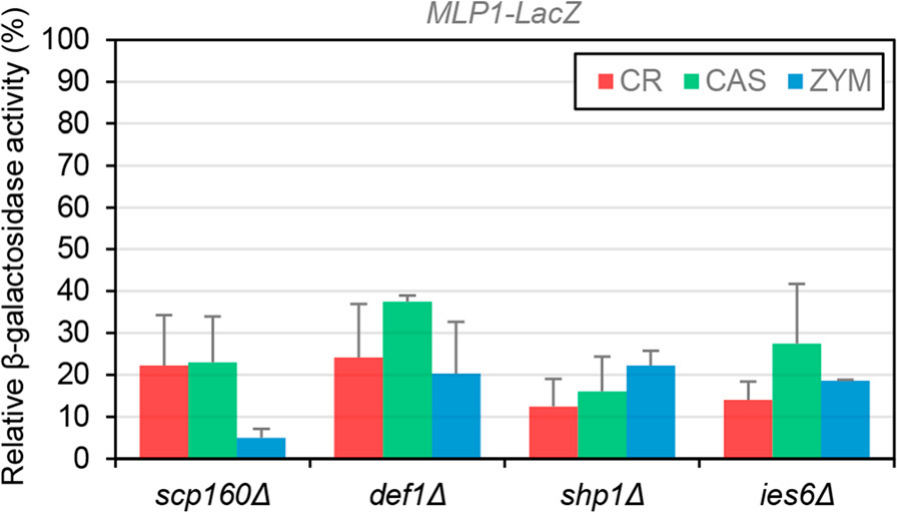
*MLP1* induction by cell wall stress is reduced in some mutants belonging to the CWM group. Expression of *pMLP1-LacZ* was measured in wild-type (BY4741) and *scp160*∆, *def1*∆, *sph1*∆, *ies6*∆ mutants growing during 3 h at 30 °C either in the absence or in the presence of CR (30 μg/ml), CAS (15 ng/ml) or ZYM (0.8 U/ml). Relative β-galactosidase activity respect to the wild-type strain (100 % of activity) for each condition is presented. Three independent experiments were carried out to calculate means and standard deviations

Chemogenomic and transcriptional profiles provide a global perspective of the processes necessary to maintain yeast cell wall homeostasis upon damage. Thus, we compared the transcriptional profiles of yeasts growing in the presence of CR, ZYM and CAS [13, 14, 68] with the hypersensitivity profiles described here finding a lack of correlation between gene expression and functional profiles, as previously described in a wide range of conditions [17, 69]. This lack of correlation could be consequence of gene functional redundancy and/or to further levels of post-translational control. Interestingly, some of the genes identified in both approaches correspond to genes functionally very relevant for the cell wall stress adaptive response like *SLT2*, the MAPK of the CWI pathway, the phosphatase *PTP2* that regulates this pathway, *FKS1* (encoding the β-1,3 GS), *CRH1* (encoding the transglycosilase responsible for the glucan-chitin crosslinking) and *DFG5* (encoding a GPI-anchored membrane protein required for cell wall biogenesis). The possibility that cell wall stress hypersensitivity could be related to the role of the corresponding gene in the development of adequate CWI transcriptional adaptive responses led us to uncover those mutants within the set of 28 strains hypersensitive to all three conditions (CAS, ZYM and CR) in which adaptive transcriptional response to the three compounds would be blocked. In a previous report, we developed a large-scale screening to identify genes required for transcriptional activation under ZYM treatment using a reporter system based on the expression levels of the *MLP1* gene [53]. *MLP1* shows low basal gene expression levels, but it is highly expressed under cell wall stress, and this induction is largely dependent on Slt2 and Rlm1 [70]. 9 out of the 28 strains hypersensitive to all three conditions were identified in that screening. Then, we measured *MLP1* expression in these mutants after treatment with CAS (15 ng/ml, 3 h), ZYM (0.8 U/ml, 3 h) and CR (30 μg/ml, 3 h). As expected, deletion of *SLT2* and *BCK1* caused the blockade of *MLP1* induction in the three conditions. Interestingly, we found four additional mutants, *scp160*Δ, *def1*Δ, *shp1*Δ and *ies6*Δ, showing a severe impairment of reporter induction by the three stresses (Fig. 4). These results suggest that reduced fitness of these mutants, under situations which compromise cell wall integrity, is due to a defective transcriptional adaptive response and identify *SCP160*, *DEF1*, *SHP1* and *IES6* as functional elements required for regulation of gene expression through the CWI pathway. Thus, chemical-genomic and genome-wide gene expression profiling provide complementary approaches to better understand the network of complex processes involved in the maintenance of the cellular homeostasis under cell wall threatening.

### Identification of a common signature of cell wall maintenance (CWM)-related genes

**Fig. 5:**
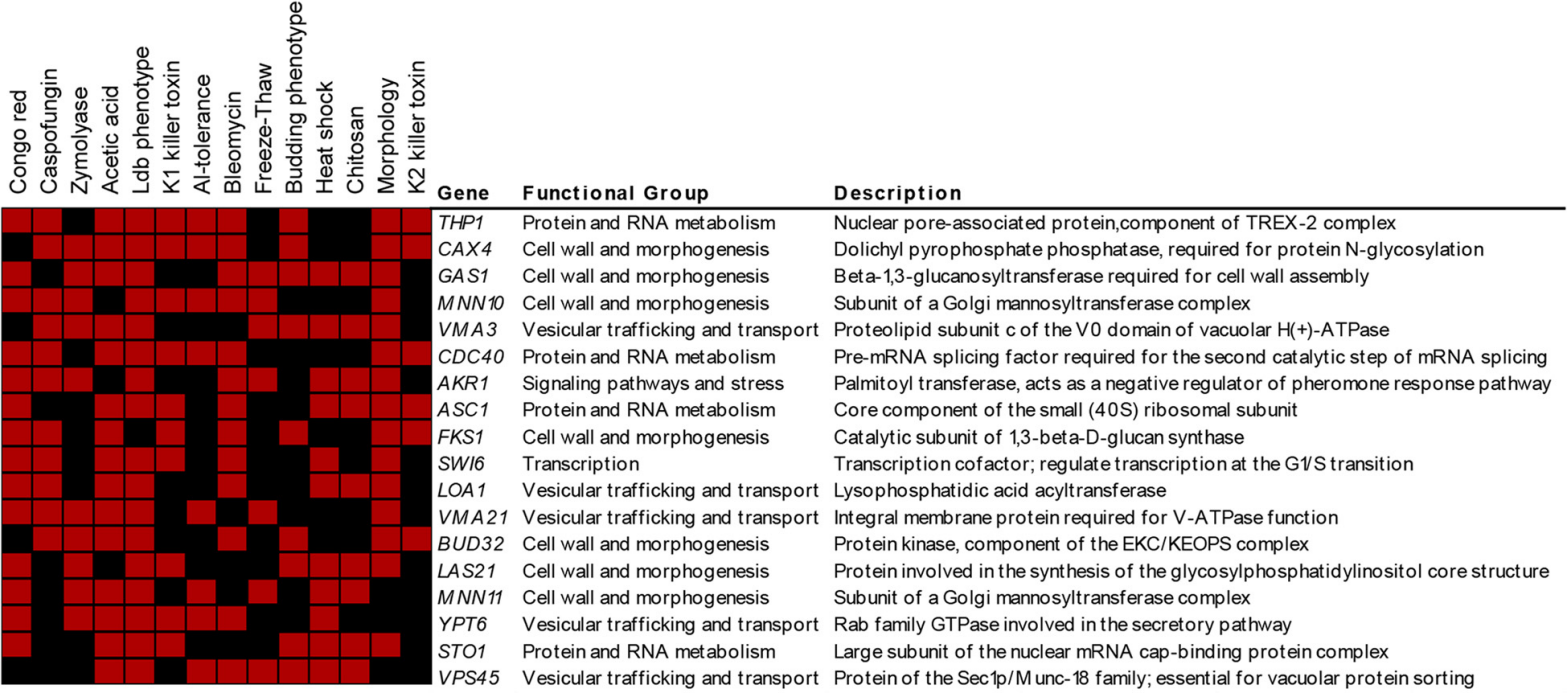
Comparison of data from CR, CAS, ZYM and published cell integrity related phenotypic screenings. The complete gene set of 636 genes identified in our screenings was compared to data from the following published large-scale phenotypic screenings: sensitivity against K1 and K2 killer toxins which bind to different cell wall receptors, ultimately forming lethal pores in the plasma membrane [27, 86]; chitosan, a deacetylated derivative of chitin which eventually causes plasma membrane stress accompanied by structural changes in the cell wall [87]; freeze-thaw or heat stress, which have been related to defects in cell wall biogenesis or assembly [88, 89]; aluminum, the small glycopeptide antibiotic bleomycin or acidic conditions, for which a proper cell wall maintenance and architecture appears to play an important protective role [90–92]; detection of low dye binding (ldb) phenotypes associated with altered mannoprotein-linked oligosaccharides located in the outer layer of the cell wall [93]; altered budding pattern [94] and morphological phenotypes [95]. A graphical representation of this comparison is shown. Vertical axis corresponds to the different conditions analyzed, and genes whose deletion cause a phenotype of hypersensitivity (labelled in *red*) in at least eight of the conditions analyzed are represented in the horizontal axis. *Black* indicates absence of hypersensitivity phenotype

One of our goals was to identify a core of key genes required for yeast survival under different cell wall stresses. These genes could be considered potential candidates as antifungal targets, and the corresponding mutant strains as biosensors to detect active molecules on the fungal cell wall. As detailed above, comparison of our phenotypic genomic profiles uncovered a core of 28 genes whose deletion renders strains hypersensitive to treatment with the three compounds tested (Table 1). Since these compounds interfere directly with the cell wall by different mechanisms, this set of genes would contain essential common functions required to withstand cell wall damage independently of the nature of the damage. Complementary, we have compared our complete set of 636 genes with data from a selected group of previously published large-scale phenotypic screenings under conditions that directly or indirectly compromise cell integrity (Fig. 5).

From this comparative analysis, we found that 75 % of the mutants with reduced tolerance to CAS, ZYM or CR showed altered fitness in at least one of the conditions under comparison (data not shown). This analysis allowed us to define a group of 18 genes common to more than 60 % of the conditions examined (Fig. 5), excluding the genes previously assigned to the group of multidrug resistance genes. All these genes, except *STO1*, *ASC1* and *VPS45*, were also identified in at least two of our screenings and three genes (*MNN10*, *AKR1* and *VMA21*) were included in the core of strains hypersensitive to CR, ZYM and CAS. Similarly to the core of 28 genes whose deletion renders strains hypersensitive to treatment with the three compounds tested (Table 1), this group included functions primarily related to cell wall biogenesis, transcription, protein and RNA metabolism and vesicular trafficking/transport.

On the basis of these results, we propose to define a common set of 43 genes as “cell wall maintenance (CWM)” signature. This signature would include the 28 genes identified as positive in our three screenings together with the additional 15 genes identified by clustering with other conditions related to cell integrity. As proof of its relevance in the cell wall homeostasis, between 72–78 % of mutants included in the CWM group exhibited medium or high sensitivity levels (class 2 and 3) to each of the tested compounds in comparison with the 53–58 % obtained when the complete set of positive hits was considered (Table 2).

**Table 2:** Cell wall maintenance (CWM)-related genes

		Hypersensitivity levels			Sorbitol-remediable hypersensitivity		
ORF	Gene	CR	CAS	ZYM	CR	CAS	ZYM
*YML008C*	*ERG6*	2	2	1	N	N	N
*YBL058W*	*SHP1*	2	3	2	Y	Y	Y
*YAL023C*	*PMT2*	3	1	2	Y	Y	Y
*YDR245W*	*MNN10*	1	3	3	Y	N	N
*YNL084C*	*END3*	3	2	1	N	N	Y
*YOR035C*	*SHE4*	3	3	3	Y	Y	Y
*YGL173C*	*KEM1*	1	1	1	N	N	N
*YKL054C*	*DEF1*	3	2	3	Y	N	N
*YJL080C*	*SCP160*	2	1	1	N	N	N
*YDL006W*	*PTC1*	3	2	2	Y	N	N
*YDR264C*	*AKR1*	1	2	3	N	N	N
*YDR477W*	*SNF1*	2	2	2	N	Y	N
*YHR030C*	*SLT2*	3	3	3	Y	Y	Y
*YJL095W*	*BCK1*	3	2	3	Y	Y	Y
*YCR020W-B*	*HTL1*	3	2	3	Y	N	N
*YBL093C*	*ROX3*	2	2	3	N	N	N
*YJL115W*	*ASF1*	3	2	1	Y	N	N
*YOL148C*	*SPT20*	1	1	2	N	N	N
*YEL044W*	*IES6*	1	1	1	N	N	N
*YJL204C*	*RCY1*	3	2	1	Y	N	N
*YLL043W*	*FPS1*	1	2	2	Y	Y	Y
*YLR138W*	*NHA1*	2	3	3	Y	Y	Y
*YOR332W*	*VMA4*	2	2	1	N	N	N
*YKL080W*	*VMA5*	3	2	3	Y	N	N
*YGR020C*	*VMA7*	2	2	2	Y	N	N
*YGR105W*	*VMA21*	2	2	2	N	N	N
*YHR060W*	*VMA22*	2	2	2	Y	N	N
*YKL119C*	*VPH2*	3	2	1	Y	N	N
*YOL072W*	*THP1*	1	1	─	N	N	─
*YGR036C*	*CAX4*	─	2	2	─	N	N
*YMR307W*	*GAS1*	2	─	3	Y	─	N
*YEL027W*	*VMA3*	─	2	1	─	N	N
*YDR364C*	*CDC40*	1	1	─	Y	Y	─
*YMR116C*	*ASC1*	2	─	─	Y	─	─
*YLR342W*	*FKS1*	3	2	─	Y	Y	─
*YLR182W*	*SWI6*	2	2	─	Y	Y	─
*YPR139C*	*LOA1*	1	1	─	N	N	─
*YGR262C*	*BUD32*	─	2	3	─	N	N
*YJL062W*	*LAS21*	3	─	2	Y	─	N
*YJL183W*	*MNN11*	3	─	2	Y	─	N
*YLR262C*	*YPT6*	3	─	2	N	─	N
*YMR125W*	*STO1*	1	─	─	N	─	─
*YGL095C*	*VPS45*	─	─	─	─	─	─

Low, medium and high levels of hypersensitivity are indicated as 1, 2, and 3, respectively. Cell growth similar to the wild-type strain is shown as "─". Sorbitol-remediable and non-remediable phenotypes are denoted with "Y" and "N", respectively

Sorbitol provides osmotic stabilization to mutants suffering from defects in the cell wall integrity and prevents cell lysis caused by a weakened cell wall. Therefore, phenotypical remediation of temperature sensitivity by sorbitol has been related to cell wall function deficiency [71, 72]. Based on this, we evaluated if addition of sorbitol 0.8 M to the cell culture was able to rescue the hypersensitivity phenotypes of the 43 mutants included in the “CWM” signature (Table 2 and Additional file 3). Addition of sorbitol resulted in remediation of 62 %, 31 % and 23 % of the mutants hypersensitive to CR, CAS and ZYM, respectively (Table 2). These results support a direct osmotic stabilization of cell wall integrity defects in these mutants. The highest percentage of remediation for CR phenotypes suggests a more direct effect of this compound on cell wall integrity, having apparently more impact on cellular osmotic stability. The absence of remediation in the rest of mutants indicates that osmotic support is not enough to revert their phenotypes, probably because the nature of the damage prevents cell growth independently of an osmotic stabilization or because in addition to the cell wall integrity, other cellular functions, such as signal transduction or cytoskeleton associated functions, are impaired in these mutants.

### Caspofungin resistant strains: association with lipid environment

Echinocandins are the most recent addition to the antifungal arsenal and have rapidly become essential in the treatment of fungal infections refractory to traditional therapies. They are cyclic lipopeptide molecules that inhibit the β-1,3-GS activity resulting in the inability of the fungus to build β-1,3-D-glucan chains. GS is composed of at least two subunits; a putative catalytic subunit (encoded by *FKS1* and *FKS2*) and a regulatory subunit encoded by *RHO1* (for a review see [73–75]). Although extensively studied, important gaps remain about the echinocandins mechanism of action. CAS is one of the clinically introduced echinocandins, for which genetic screens of *S. cerevisiae* deletion libraries for resistant mutants has been pursued as a method to identify cellular functions affected by this drug [28, 29, 76]. Considering the clinical significance of this resistance and the low degree of overlaping genes identified in the previous studies, we performed a screening to identify mutants resistant to CAS using the same strategy utilized for the selection of hypersensitive mutants but in the presence of 60, 90 and 120 ng/ml of the drug, corresponding to 1.5, 2.25 and 3-fold higher than the minimal inhibitory concentration (MIC) (see Methods). 25 mutant strains showed enhanced CAS resistance, including 13 genes that had not been previously identified, thereby increasing significantly the number of genes associated with this phenotype (Table 3). One of the mutants identified was *wsc1*Δ, deleted in one of the sensors of the CWI pathway and defective in Rho1-dependent activation of GS [44]. In the absence of Wsc1, the MAPK Slt2 is not activated in the presence of CAS [43] and therefore the transcriptional response to this drug is severely impaired [36].

**Table 3:** Genes whose deletion causes enhanced resistance to caspofungin

ORF	Gene			Functional group	Description
*YBR105C*	*VID24*	•	CR	carbohydrate metabolism	GID Complex regulatory subunit involved in response to glucose through interactions with complex member Vid28
*YCL061C*	*MCR1*			cell cycle	S-phase checkpoint protein required for DNA replication
*YJL157C*	*FAR1*			cell cycle	Inhibitor of Cdc28-Cln1 and Cdc28-Cln2 kinase complexes involved in cell cycle arrest for matin
*YOR008C*	*SLG1*	•		cell wall	Sensor-transducer of the stress-activated CWI kinase pathway involved in maintenance of cell wall integrity
*YBR026C*	*ETR1*			lipid metabolism	2-enoyl thioester reductase with a probable role in fatty acid synthesis
*YBR036C*	*CSG2*	•	CR	lipid metabolism	ER membrane protein required for mannosylation of inositol phosphorylceramide (sphingolipids biosynthesis)
*YCR034W*	*ELO2*	•	CR	lipid metabolism	Fatty acid elongase involved in sphingolipid biosynthesis
*YGR263C*	*SAY1*			lipid metabolism	Sterol deacetylase, component of the sterol acetylation/deacetylation cycle along with Atf2
*YLR056W*	*ERG3*	•		lipid metabolism	C-5 sterol desaturase, catalyzes the introduction of a C-5(6) double bond into episterol (ergosterol biosynthesis)
*YLR372W*	*ELO3*	•	CR	lipid metabolism	Elongase involved in fatty acid and sphingolipid biosynthesis
*YNL156C*	*NSG2*			lipid metabolism	Protein involved in regulation of sterol biosynthesis stabilizing Hmg2 protein
*YPL057C*	*SUR1*	•	CR	lipid metabolism	Mannosylinositol phosphorylceramide (MIPC) synthase catalytic subunit (sphingolipids biosynthesis)
*YPL118W*	*MRP51*		CR	mitochondrial	Mitochondrial ribosomal protein of the small subunit
*YBR048W*	*RPS11B*			protein metabolism	Protein component of the small (40S) ribosomal subunit
*YBL075C*	*SSA3*			stress	ATPase, member of the heat shock protein 70 (*HSP70*) family, involved in protein folding and the response to stress
*YNL080C*	*EOS1*	•		stress	Protein involved in N-glycosylation
*YOR061W*	*CKA2*	•	CR	transcription	Alpha' catalytic subunit of casein kinase 2 (CK2) a Ser/Thr protein kinase with roles in cell growth and proliferation
*YNL323W*	*LEM3*	•		transport	Protein of the plasma membrane and ER involved in translocation of phospholipids across the plasma membrane
*YBL029W*				unknown	Non-essential protein of unknown function
*YBR071W*				unknown	Protein of unknown function, may be regulated by the cell cycle and/or cell wall stress
*YDR360W*	*OPI7*			unknown	Dubious open reading frame, partially overlaps verified gene *VID21/YDR359C*
*YEL025C*				unknown	Putative protein of unknown function
*YOR024W*		•		unknown	Dubious open reading frame
*YOR118W*		•	CR	unknown	Protein of unknown function
*YPR038W*	*IRC16*			unknown	Dubious open reading frame, partially overlaps verified gene *ERV2/YPR037C*

Those deletion mutants that have been previously reported to be resistant to caspofungin are labeled with a black dot. CR indicates resistance to Congo red respect to the wild-type strain

**Fig. 6:**
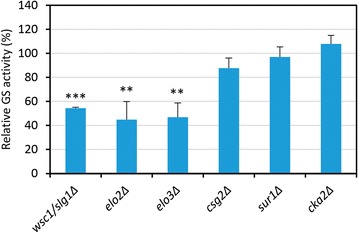
Quantification of the β-1,3-glucan synthase (GS) activity of mutants resistant to Caspofungin. Cell extracts from early log-phase cultures in YEPD medium at 30 °C of the wild-type (BY4741) and the 25 CAS resistant mutant strains were obtained, and used for enzymatic assays as described in Methods. It is shown the GS activity of the six mutants related to sphingolipid biosynthesis. *Bars* represent the percentage of GS specific activity in each mutant compared to that of the wild-type strain (100 % of GS specific activity). Data are the mean of three independent experiments. *Error bars* represent SD. Statistical analysis was carried out with a two-tailed, unpaired, Student’s *t*-test to analyze differences between the indicated mutants and the wild-type strain: **P ≤ 0.01; ***P ≤ 0.001

An important group of hyper-resistant strains was affected in genes functionally related to lipid metabolism, including genes involved in sphingolipid biosynthesis like *CSG2*, *ELO2*, *ELO3*, *CKA2* and *SUR1*, sterol biosynthesis (*SAY1*, *ERG3* and *NSG2*), fatty acid synthesis (*ETR1*) and translocation of phospholipids across the plasma membrane (*LEM3*) [77]. Yeast cells lacking *LEM3* have recently been reported to exhibit resistance to the N-glycosylation inhibitor tunicamycin in a screening to identify carriers involved in drug uptake [78]. The yeast β-1,3 GS is inhibited non-competitively by intermediates of the sphingolipid biosynthesis, dihydrosphingosine (DHS) or phytosphingosine (PHS), being *in vivo* PHS the primary GS inhibitor [79]. Accumulation of PHS/DHS might repress the interaction between Fks1 and Rho1 or alternatively alter the environment of lipid bilayer, causing inactivation of GS. In *Candida glabrata*, membrane sphingolipids also modulate echinocandin-Fks interaction, the accumulation of long chain bases (LCBs) DHS or PHS conferring resistance to CAS [76]. To evaluate the possible association between GS activity and enhanced CAS resistance, we determined *in vitro* the activity of this enzyme in the membrane fraction of the 25 mutants identified in our screening. As a result, only three of them, *wsc1*Δ, *elo2*Δ and *elo3*Δ, showed a significant decrease in the β-1,3-GS activity (Fig. 6 and data not shown) compared to the wild type strain, but still maintaining an *in vivo* GS activity enough for cell viability. A decrease in the GS activity of the *elo2*Δ mutant strain, affected in sphingolipid biosynthesis and resistant to echinocandins, has been previously described [80]. Our data show that Elo3 is also required for an adequate GS activity. However, mutants *csg2*Δ (Csg2 is required for mannosylation of inositol phosphorylceramide (IPC)), *sur1*Δ (Sur1 encodes the Mannosylinositol phosphorylceramide (MIPC) synthase catalytic subunit) and *cka2*Δ (Cka2 is involved in phosphorylation of ceramide synthase), although also involved in the synthesis of complex sphingolipids, did show levels of GS activity similar to that of the wild-type strain (Fig. 6). This observation opens the possibility that IPC accumulation or depletion of terminal complex sphingolipids might be associated to CAS interaction and resistance, but without affecting directly GS activity. This is consistent with the fact that mutations of Fks1 GS that confer resistance to echinocandins, also present a GS activity similar to that of the wild type GS [81–83]. Next, we wondered if CAS resistance correlates with resistance to other cell wall injuries. We were unable to carry out these assays with zymolyase due to the intrinsic resistance of the wild-type strain to this compound (growth was only partially reduced even in the presence of 500 U/ml). To test resistance to Congo red, we employed the spot assay protocol using plates containing 250 μg/ml of this dye. This concentration severely impaired the growth of the wild-type strain and allowed us to identify resistant mutant strains. From the 25 strains tested, 8 (32 %) manifested a resistance phenotype (Table 3). Interestingly, the five strains involved in sphingolipid biosynthesis described above were detected within this group, reinforcing the notion of the key role of sphingolipids in cell wall maintenance. These results are in agreement with the hypothesis that sphingolipids regulate stress resistance, since down-regulating sphingolipid synthesis renders cells resistant to heat, oxidative and acetic acid stresses [84].

## Conclusions

The characterization of the molecular mechanisms required for cell wall maintenance, including those governing adaptation to cell wall stress, is absolutely necessary, not only to understand the basis of relevant biological processes like cell wall construction and morphogenesis, but also for the discovery of new antifungal targets. This work contributes significantly to these objectives by relying on a chemical-genomic profiling strategy to identify functional relationships between specific genes and chemical compounds that affect yeast cell wall integrity by means of different mechanisms of action, namely CR, CAS, and ZYM.

Functional analysis of the identified mutants shows that fungal cells buffer the damage in specific components of the cell wall through multiple complementary mechanisms. Indeed, mutants affected in glucan network and organization of the cytoskeleton or chitin synthesis were especially sensitive against the chitin-binding dye CR and the β-1,3-GS inhibitor CAS, respectively whereas strains affected in protein mannosylation were hypersensitive to the three compounds but mainly to ZYM, an enzymatic complex that affects the entire cell wall. In addition to the cell wall, the most important functions uncovered included signal transduction, chromatin remodeling and transcription, RNA metabolism, vesicular transport and ion homeostasis. Although these functional groups were in common to the three different compounds assayed, specific genes/proteins were required to cope with distinct cell wall stresses, suggesting a broad versatility of the cell wall stress response network.

A limited but significant core of 28 deletion strains displayed overlaping fitness profiles for the three compounds, unraveling essential functions required to tolerate cell wall stress independently of the nature of the damage. Comparison of our phenotypical profiles with previous studies using conditions putatively affecting cell integrity, uncovered an additional cluster of 15 deletion strains, defining a common set of 43 genes named here as a “cell wall maintenance (CWM)” gene signature. Those functional groups within this signature included: a) Cell wall and morphogenesis, including genes involved in cell wall protein mannosylation and maturation (*MNN10, MNN11, PMT2, LAS21, CAX4*), cell wall remodeling (*GAS1, FKS1*) and cytoskeleton (*END3, SHE4*); b) Transcription and chromatin remodeling, including components of the RNA PolII Mediator, RSC, SAGA, INO80, SWR1 and Swi6, a component of the SBF (Swi4-Swi6) and MBF (Mbp1-Swi6) transcriptional complexes; c) Signal transduction and stress response: as expected, the MAPK Slt2 and the MAPKK Bck1 of the CWI pathway were included in this group. Additionally, the phosphatase Ptc1, the palmitoyl transferase Akr1 and the AMP-activated Ser-Thr kinase Snf1 seem to be very important for cell wall stress adaptation; d) RNA metabolism, including the RNA binding protein Scp160, required for translational efficiency of codon-optimized mRNAs in yeast; e) Ergosterol biosynthesis (*ERG6*) and f) Vesicular trafficking and transport (mainly those required for V-ATPase activity). This is one of the largest functional group identified in our chemical genomic profiles. It includes a wide range of cellular trafficking-related systems such as VPS vacuolar sorting, endosomal sorting complexes (ESCRT), SNARE complex and the V-type H + -ATPase. All these genes included in the “cell wall maintenance (CWM)” gene signature might be used as biomarkers of cell wall stress in future genomic-fitness profiles.

Elements responsible for adaptive response to specific stresses were also uncovered. For CR, the CWI pathway was the main route involved, whereas decreased tolerance to ZYM was dependent on both the CWI and HOG MAPK pathways. Intriguingly, for CAS, in addition to the CWI pathway, the complete kinase module belonging to the invasive growth MAPK pathway (Ste11, Ste7 and Kss1) was also uncovered. This observation points out to a possible connection between the CWI and the invasive growth pathways, bearing in mind that the transcriptional response to CAS is completely dependent on the CWI sensor Wsc1 [36].

The search for mutants showing increased resistance to CAS identified 25 genes associated to this phenotype, including several genes involved in the synthesis of sphingolipids and ergosterol. Interestingly, three of these mutants, *wsc1*Δ, *elo2*Δ and *elo3*Δ, presented decreased GS activity further confirming that specific lipid content affects GS activity. In contrast, some of the mutations identified affecting sphingolipids amount were associated to CAS resistance without affecting GS activity. Moreover, deletion of genes involved in sphingolipid biosynthesis also rendered cells resistant to CR, further confirming a key role for sphingolipids in global cell wall integrity maintenance under stress.

Taking into account the compensatory mechanisms triggered against cell wall defects, the knowledge provided here might be useful for identification of potential antifungal targets in a more efficient antifungal strategy by combination of two drugs, one targeting the cell wall and the other interfering with the adaptive mechanisms. In this context, our cell wall-chemogenomic profiles clearly point to the importance of the V-ATPase function for withstanding the cell wall damage caused by different cell wall interfering-compounds. In agreement, we found that simultaneous inhibition of the V-ATPase by fluconazole and of cell wall biogenesis by cell wall interfering-drugs leads to synergistic antifungal effects.

## Methods

### Yeast strains and media

Experiments were performed with the full collection of *Saccharomyces cerevisiae* strains (BY4741 background, *MAT*a; *his3*Δ-*1*; *leu2*Δ-*0*; *met15*Δ-*0*; *ura3*Δ-*0*) individually deleted in all of the ORFs identified in this organism (4786) that were replaced by the Geneticin resistance codifying KanMX4 module. This collection was provided by Euroscarf (Germany). *S. cerevisiae* was grown on YEPD medium (2 % glucose, 2 % peptone, 1 % yeast extract). When required, sorbitol (PanReac-AppliChem, Spain) 0.8 M or fluconazole (Fluka, Sigma-Aldrich, USA) were added at the specified concentrations to the culture media.

### Screening for yeast mutants hypersensitive to zymolyase (ZYM)

The full collection of yeast mutant strains were grown in 100 μl of YEPD medium in 96-well microtiter plates for 24 h at 30 °C. Then, cultures were diluted preparing a new set of plates containing 180 μl of fresh YEPD per well plus 5 μl of the original cultures. Finally, plates containing 100 μl of YEPD plus 30 U/ml of zymolyase 20 T (ImmunO™, MP Biomedicals, USA) were inoculated with 5 μl of the aforementioned plates and incubated in a static culture at 30 °C. After 24 h, growth was determined by measuring absorbance at 595 nm (A_595_) in each well using a microplate reader (Model 680, Bio-Rad, USA). Growth inhibition by ZYM for each strain was calculated relative to the A_595_ value of a wild-type strain handled in the same conditions. Three categories of hypersensitivity to ZYM were arbitrary defined consisting of those mutants with a growth rate relative to the wild type strain in the presence of the enzymatic complex about 50–26 % (class 1), 25–11 % (class 2), and ≤ 10 % (class 3). Positive hits from this chemical-genomic profile were confirmed by determination of the minimum inhibitory concentration (MIC) for ZYM (see below).

### Screening for yeast mutants hypersensitive to Congo red (CR)

The collection of yeast mutant strains were grown overnight in 96-well microtiter plates in 100 μl of YEDP medium at 30 °C. Then, cultures were diluted preparing a new set of plates containing 95 μl of YEPD per well plus 5 μl of the original cultures. From these new plates, three serial 1:10 dilutions were prepared in fresh YEPD and 5 μl of each dilution was spotted with a 6 × 8 array replica platter (Sigma-Aldrich, USA) onto solid YEPD and YEPD plus 50 μg/ml of Congo red (Merck Millipore, USA) plates. After 40, 70 and 100 h of incubation at 30 °C, the level of growth for each mutant strain was recorded. Hypersensitivity to CR was determined comparing the growth on CR-containing plates respect to the wild-type (BY4741) and the *slt2*∆ strains. The *slt2*∆ mutant was selected since its hypersensitivity to cell wall stress is well known [13]. Mutant strains hypersensitive to CR were arbitrarily grouped in three categories taking as reference the behavior of *slt2*∆ mutant grown at the same conditions. Class 3 includes mutant strains more sensitive to CR than *slt2*∆; class 2 was assigned when the sensitivity to CR was similar to *slt2*∆; and class 1 comprises strains with reduced growth respect to the wt strain but higher than *slt2*∆.

### Screening for yeast mutants hypersensitive and resistant to caspofungin (CAS)

The complete collection of yeast mutant strains were grown in 96-well microtiter plates in 100 μl of YEDP medium for 24 h at 30 °C. Afterwards, for the hypersensitivity screening, 2 μl of these cultures were inoculated in a new set of 96-well microtiter plates containing 98 μl of YEPD and 98 μl of YEPD plus 10, 20 and 30 ng/ml of caspofungin (provided by MSD Research Laboratories, USA). YEPD containing 60, 90 and 120 ng/ml of CAS was used to identify mutants resistant to this drug. After 46 h of static incubation at 30 °C, growth was determined by measuring the absorbance at 595 nm in each well using a microplate reader (Model 680, Bio-Rad, USA). The level of sensitivity of each mutant to each concentration of CAS tested was calculated as the following ratio: absorbance in YEPD plus CAS/absorbance in YEPD. A mutant strain was considered hypersensitive to the drug when this ratio was ≤0.5 (growth decrease ≥50 %). Positive hits were confirmed performing a new screening following the same strategy as described above. Eventually, three categories of hypersensitivity to CAS were defined: mutant strains hypersensitive at 10, 20 and 30 ng/ml of CAS (class 3), hypersensitive at 20 and 30 ng/ml (class 2), and hypersensitive only at 30 ng/ml (class 1). On the other hand, a mutant strain was considered resistant if the ratio (absorbance in YEPD plus caspofungin)/(absorbance in YEPD), for the three concentrations tested (60, 90 and 120 ng/ml) was higher than 1.5 (growth increase ≥50 %).

### Minimal inhibitory concentration (MIC) assays

ZYM, CR and CAS sensitivity assays were performed in 96-well microtiter plates, preparing two-fold serial dilutions of zymolyase 20 T, Congo red and caspofungin to give concentrations ranging from 125 to 0.25 U/ml, 1920 to 1.9 μg/ml and 1 to 0.001 μg/ml respectively, in a final volume of 150 μl of YEPD. Each well was inoculated with approximately 10^4^ cells from an exponentially growing culture. Plates were incubated at 30 °C and cell growth was determined after 24 and 48 h by measuring the absorbance at 595 nm using a microplate reader. Sorbitol-remediable hypersensitivity assays were carried out in the same way but in presence of sorbitol 0.8 M. Cell growth remediation was considered when the MIC was increased at least in one order of magnitude in the presence of the osmotic support.

### Hypersensitivity assays to fluconazol and cell wall-interfering compounds

The assays were performed in a final volume of 150 μl of YEPD in 96-well microtiter plates. Cell growth of wild-type yeast cells, treated with different amounts of fluconazole (ranging from 16 to 128 μg/ml) either in the absence or in the presence of sublethal concentrations of CR, CAS or ZYM (30 μg/ml, 15 ng/ml and 64 U/ml, respectively), was determined after 24 h of incubation at 30 °C by measuring the absorbance at 595 nm.

### β-galactosidase assays

β-galactosidase assays were performed using crude cellular extracts from exponentially growing yeast cells transformed with the centromeric plasmid *pMLP1-LacZ* (promoter of *MLP1* fused to *LacZ*) as previously described [14].

### Enzyme preparation and in vitro β-1,3-Glucan synthase assay

Cell extracts and GS assays were performed essentially as described previously [85]. Membrane enzyme extracts were obtained from early log phase cells grown in YEPD medium at 30 °C. GS assay mixture contained 5 mM UDP-D-[^14^C]glucose (4 × 10^4^ cpm/200 nmoles), 150 μM GTPγS and membrane enzyme (20-40 μg of protein) in a total volume of 40 μl. All GS assays were carried out in duplicate and data for each strain were calculated from at least three independent cell cultures. Statistical data analysis were done using the GraphPad V5 software.

### Data analysis

Genes were assigned to functional categories using the yeast Gene Ontology (GO) tool “GO Slim Mapper” and further manually refined according to annotations of the BIOBASE Knowledge Library Proteome and the *Saccharomyces* Genome Database (SGD). Statistically significant functional groups, appearing in our lists at a frequency greater than it would be expected by chance, were obtained by means of Gene Ontology (GO) tool “GO Term Finder” (version 0.83) using default settings at the SGD link with GO category “Process”, *p*-value ≤ 0.01 and False Discovery rate (FDR) option. The Heat map shown in Fig. 5 was obtained using the Multiexperiment Viewer (MeV 4.9) software.

### Deposition of data

Not applicable

### Availability of supporting data

Other information is provided as supplementary files.

## Additional files

Additional file 1:
**Setting up of CAS, ZYM and CR hypersensitivity assays.** Exponentially growing cells in YEPD medium of wild-type (BY4741) and the indicated mutant strains were inoculated in fresh YEPD medium containing different concentrations of CAS (a) or ZYM (b) in 96-well microtiter plates and incubated at 30 °C for 48 h. Cell growth was determined by measurement of absorbance at 595 nm (A_595_). (c) 1:10 serial dilutions of exponentially growing wild-type and mutant strains were spotted onto YEPD plates and YEPD plates supplemented with 50 μg/ml of CR and incubated at 30 °C for 3 days. (PDF 89 kb) Additional file 2:
***Saccharomyces cerevisiae*** **mutant strains hypersensitive to Congo red, caspofungin and zymolyase classified by functional groups.** (XLS 121 kb) Additional file 3:
**Osmotic remediation by sorbitol of ZYM, CR and CAS hypersensitivity of mutant strains belonging to the “cell wall maintenance (CWM)” group.** Minimal Inhibitory concentration (MIC) assays were carried out in 96-well microtiter plates as detailed in Methods either in the absence or in the presence of 0.8 M sorbitol. (PDF 1105 kb)

## Abbreviations

CAS: Caspofungin
CR: Congo red
CWI: Cell wall integrity
CWM: Cell wall maintenance
DHS: Dihydrosphingosine
GO: Gene ontology
GS: β-1,3 glucan synthase
HOG: High-osmolarity glycerol
IPC: Inositol phosphorylceramide
MAPK: Mitogen-activated protein kinase
MDR: Multidrug resistance
MIC: Minimal inhibitory concentration
PHS: Phytosphingosine
SGD: *Saccharomyces* Genome Database
ZYM: Zymolyase
