# Supplementary figures and images for: Genomic profiling of fungal cell wall-interfering compounds: identification of a common gene signature
Source: BMC Genomics. 2015 Sep 5;16(1):683. doi: 10.1186/s12864-015-1879-4 (PMC4560923; doi:10.1186/s12864-015-1879-4)

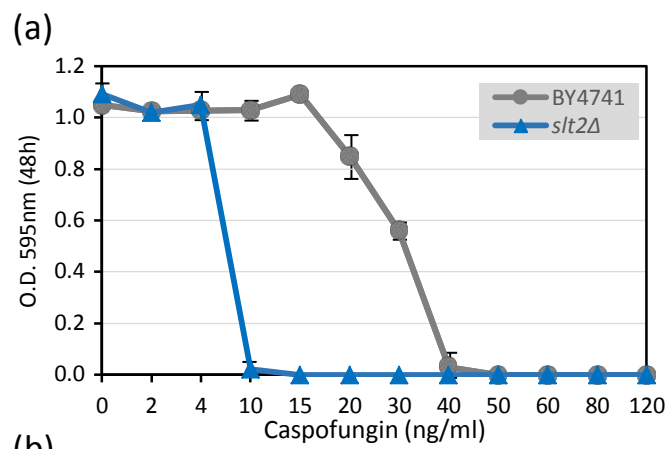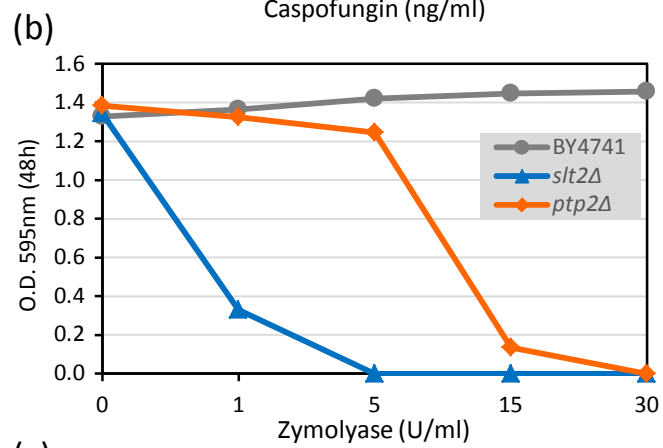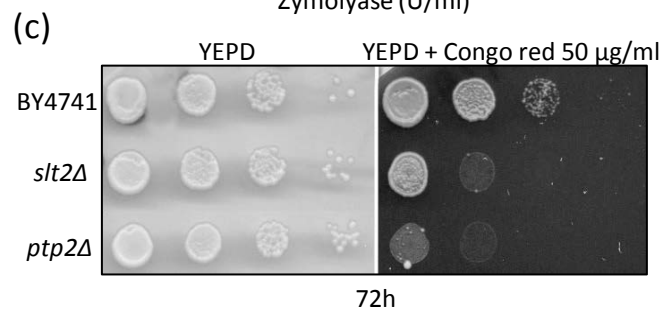

Supplement: Additional file 1: — Setting up of CAS, ZYM and CR hypersensitivity assays. Exponentially growing cells in YEPD medium of wild-type (BY4741) and the indicated mutant strains were inoculated in fresh YEPD medium containing different concentrations of CAS (a) or ZYM (b) in 96-well microtiter plates and incubated at 30 °C for 48 h. Cell growth was determined by measurement of absorbance at 595 nm (A595). (c) 1:10 serial dilutions of exponentially growing wild-type and mutant strains were spotted onto YEPD plates and YEPD plates supplemented with 50 μg/ml of CR and incubated at 30 °C for 3 days. (PDF 89 kb) [file 12864_2015_1879_MOESM1_ESM.pdf]
