# Supplementary material for: Genomic profiling of fungal cell wall-interfering compounds: identification of a common gene signature
Source: BMC Genomics. 2015 Sep 5;16(1):683. doi: 10.1186/s12864-015-1879-4 (PMC4560923; doi:10.1186/s12864-015-1879-4)
Supplement: Additional file 3: — Osmotic remediation by sorbitol of ZYM, CR and CAS hypersensitivity of mutant strains belonging to the “cell wall maintenance (CWM)” group. Minimal Inhibitory concentration (MIC) assays were carried out in 96-well microtiter plates as detailed in Methods either in the absence or in the presence of 0.8 M sorbitol. (PDF 1105 kb) [file 12864_2015_1879_MOESM3_ESM.pdf]

*fks1*Δ and *nha1*Δ

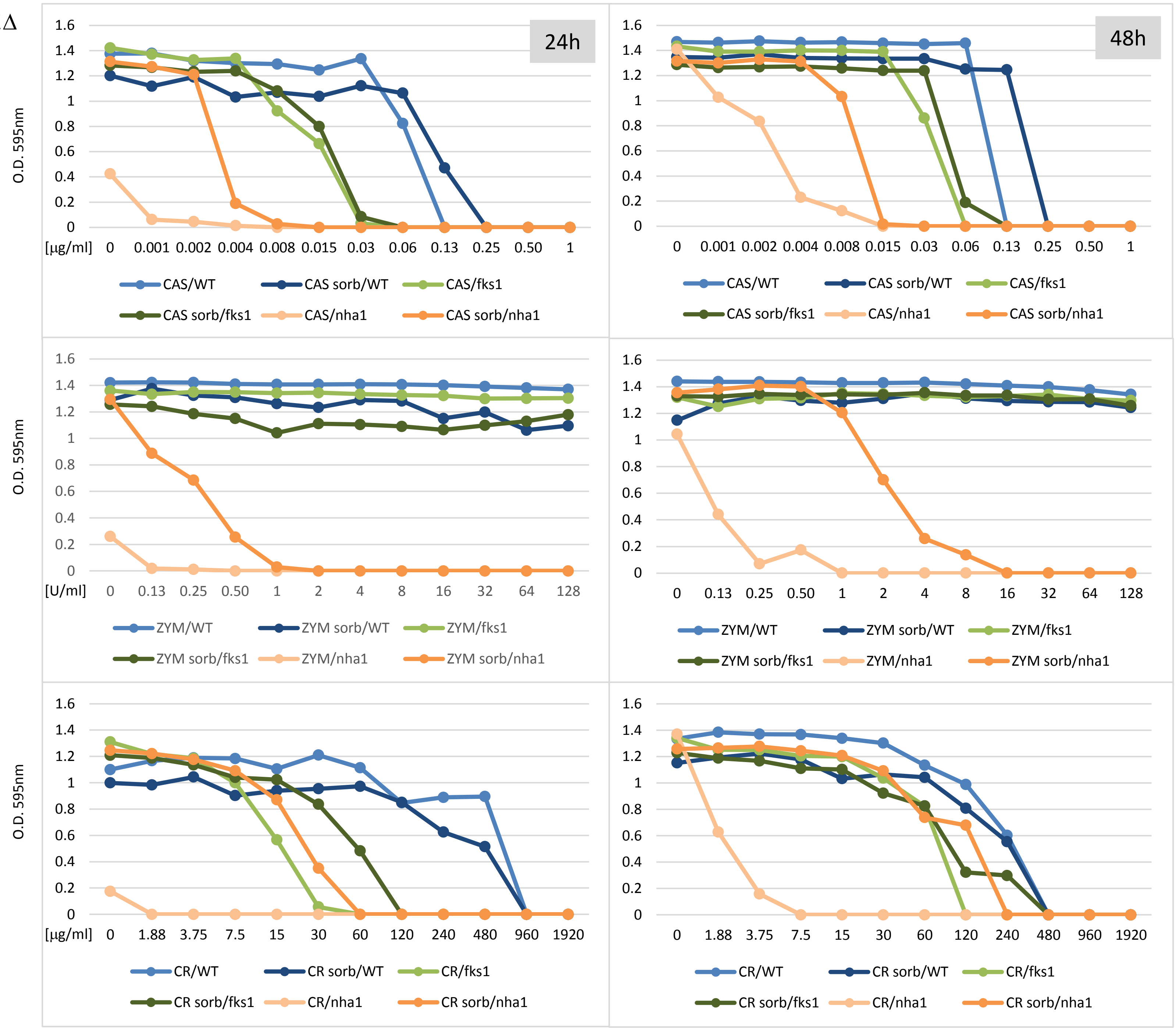

*asc1*Δ and *sto1*Δ

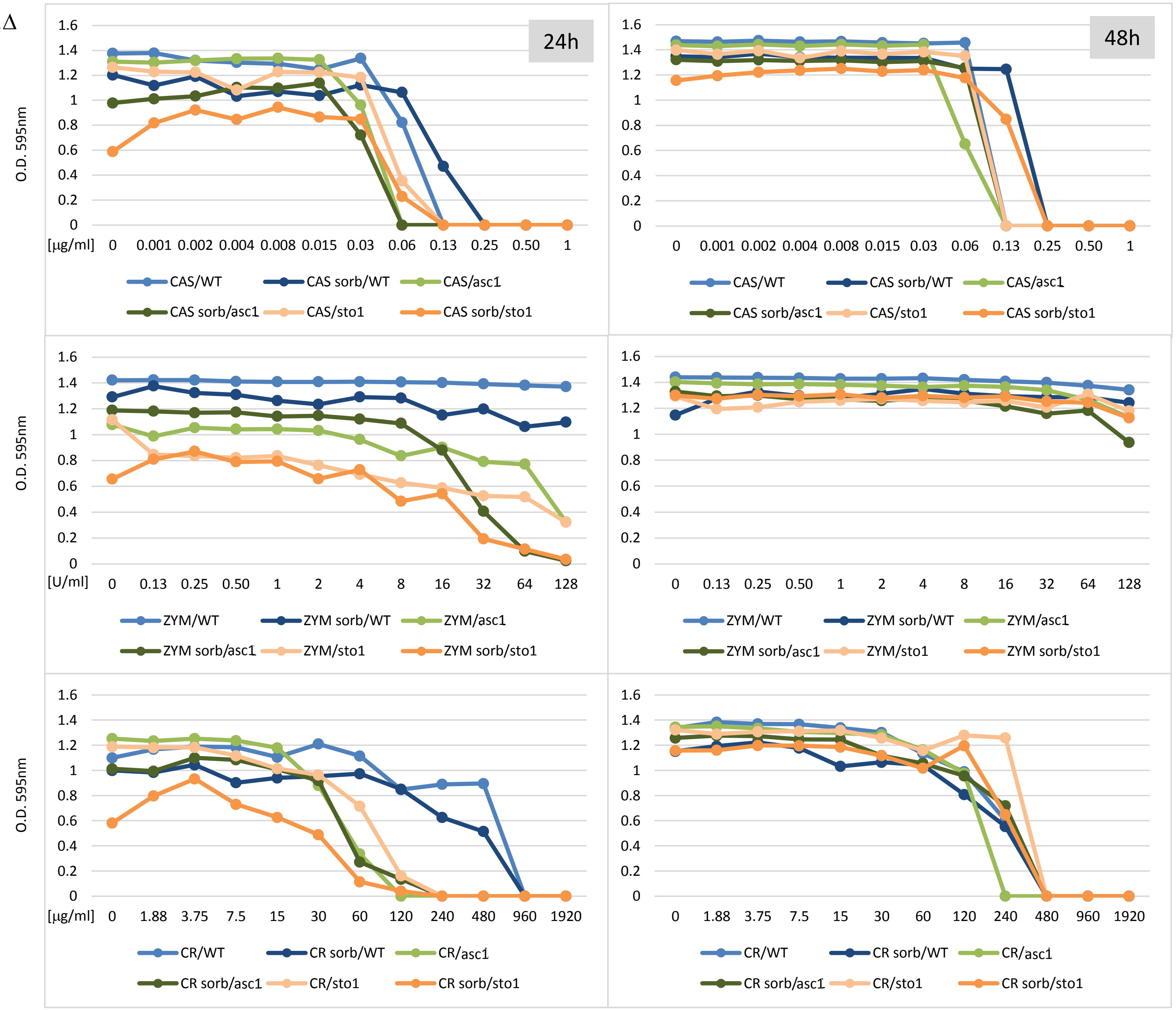

*htl1*Δ and *slt2*Δ

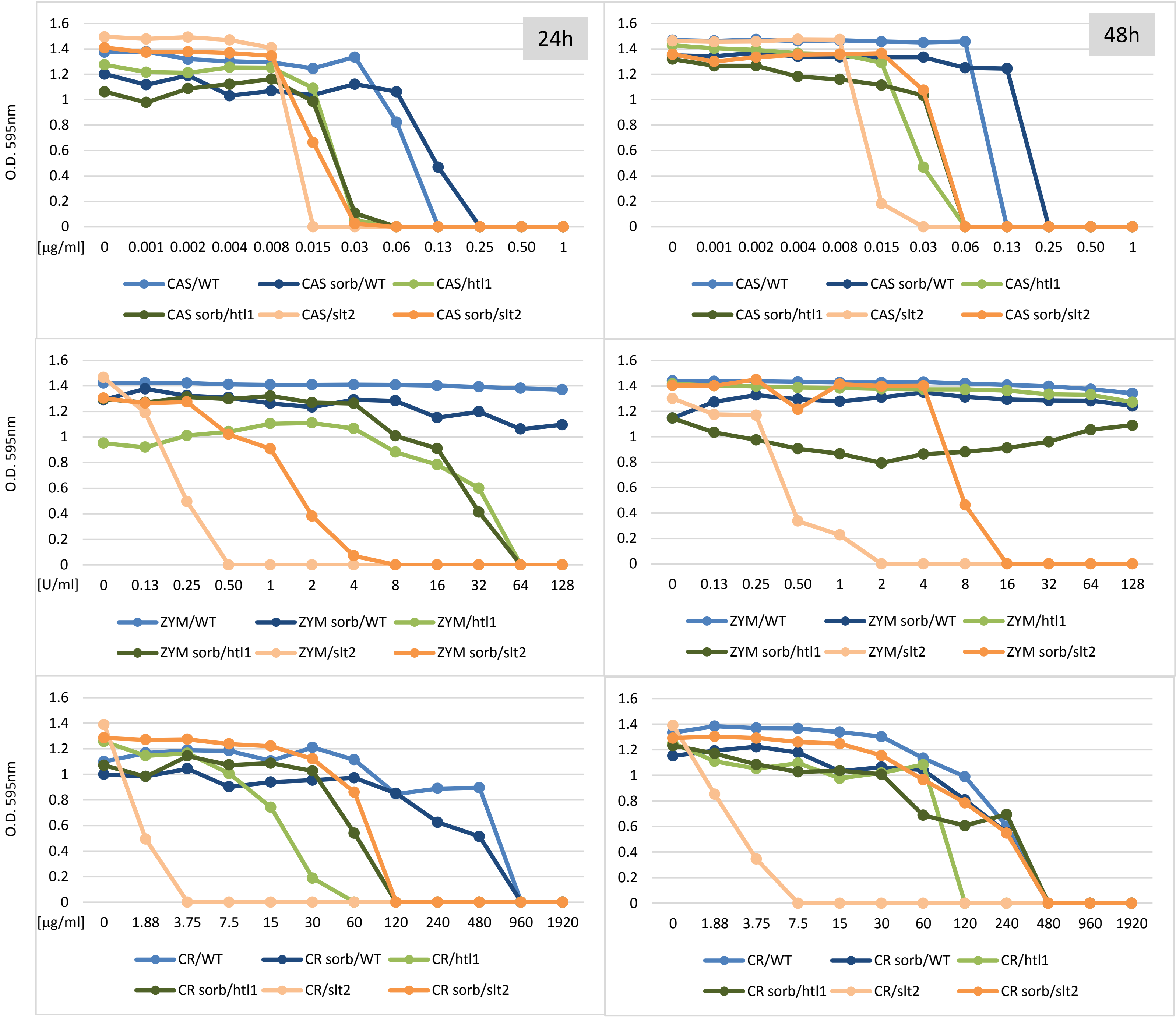

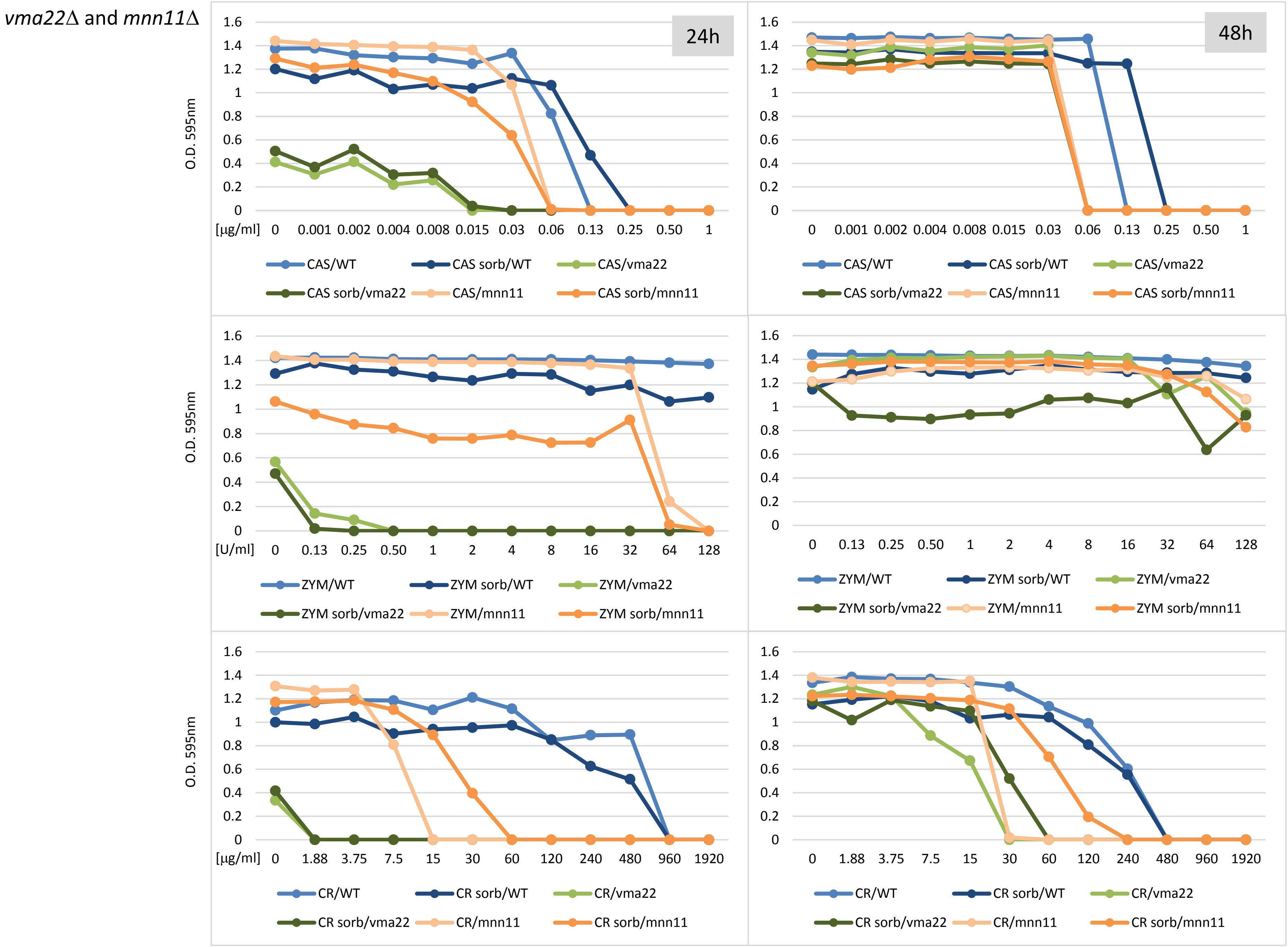
vma22Δ and mnn11Δ
24h
48h
O.D. 595nm
1.6
1.4
1.2
1
0.8
0.6
0.4
0.2
0
[μg/ml]
0 0.001 0.002 0.004 0.008 0.015 0.03 0.06 0.13 0.25 0.50 1
CAS/WT
CAS sorb/WT
CAS/vma22
CAS sorb/vma22
CAS/mnn11
CAS sorb/mnn11
[U/ml]
0 0.13 0.25 0.50 1 2 4 8 16 32 64 128
ZYM/WT
ZYM sorb/WT
ZYM/vma22
ZYM sorb/vma22
ZYM/mnn11
ZYM sorb/mnn11
[μg/ml]
0 1.88 3.75 7.5 15 30 60 120 240 480 960 1920
CR/WT
CR sorb/WT
CR/vma22
CR sorb/vma22
CR/mnn11
CR sorb/mnn11

*rcy1*Δ and *vma21*Δ

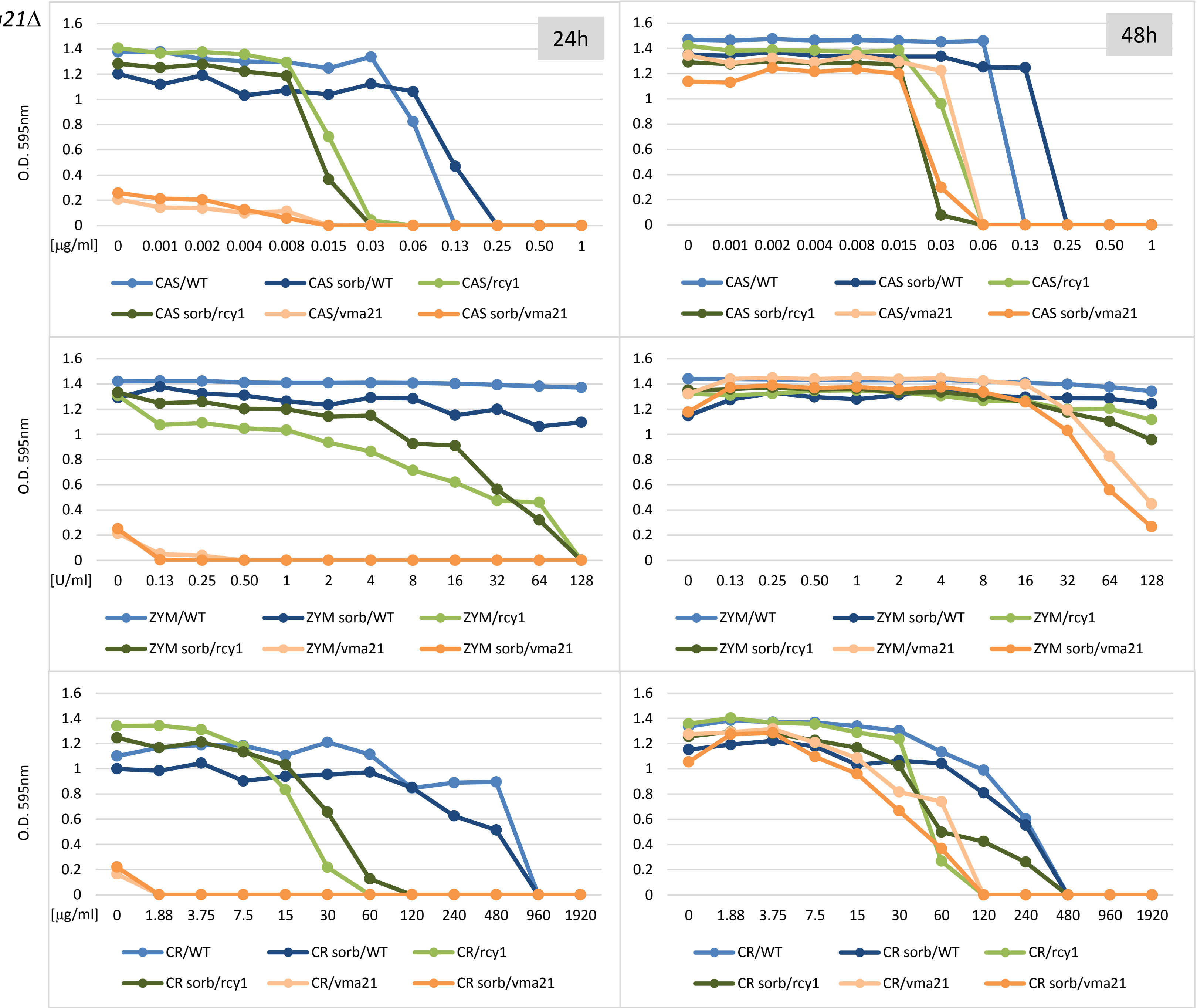

*kem1*Δ and *swi6*Δ

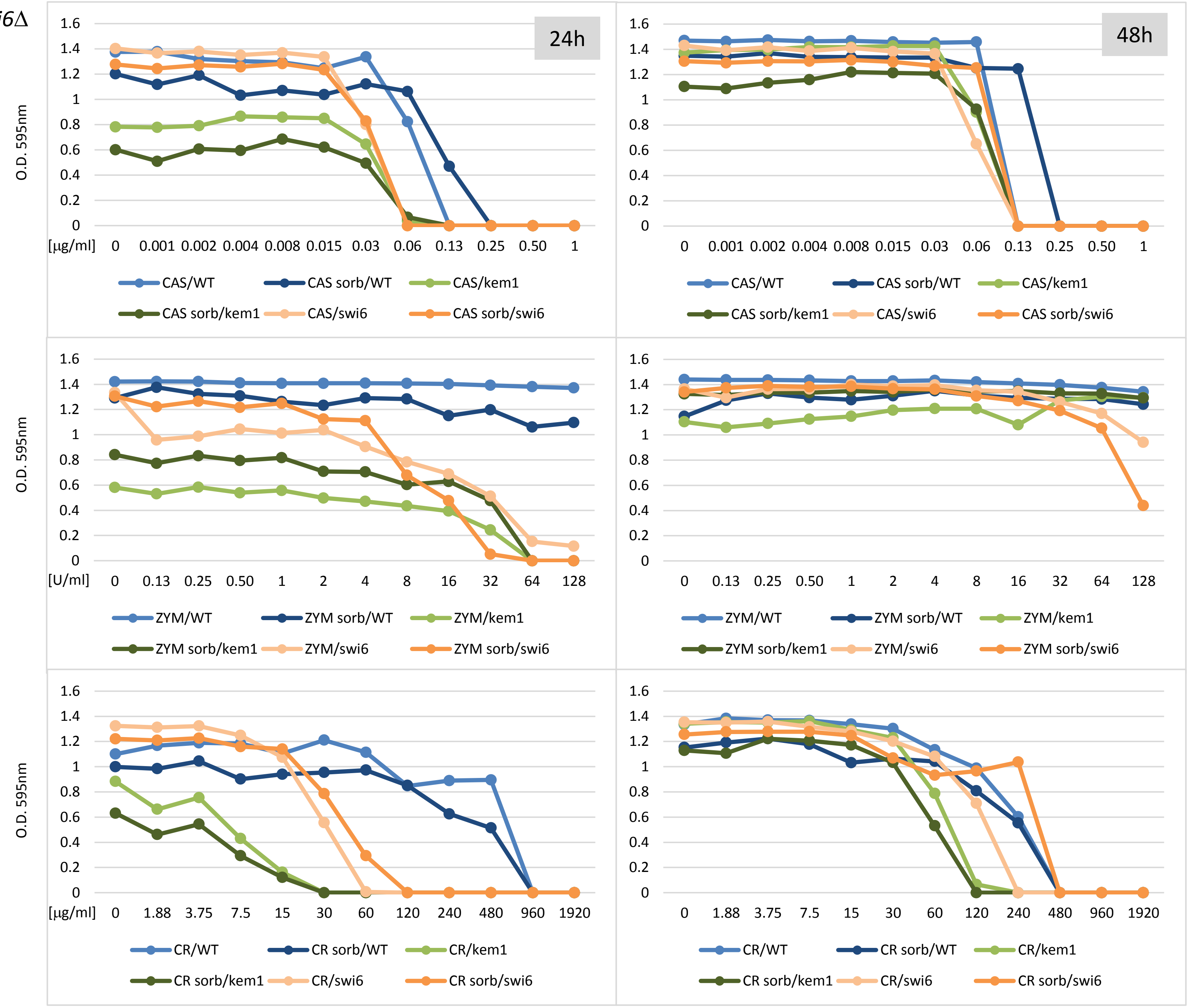

*cax4*Δ and *vma7*Δ

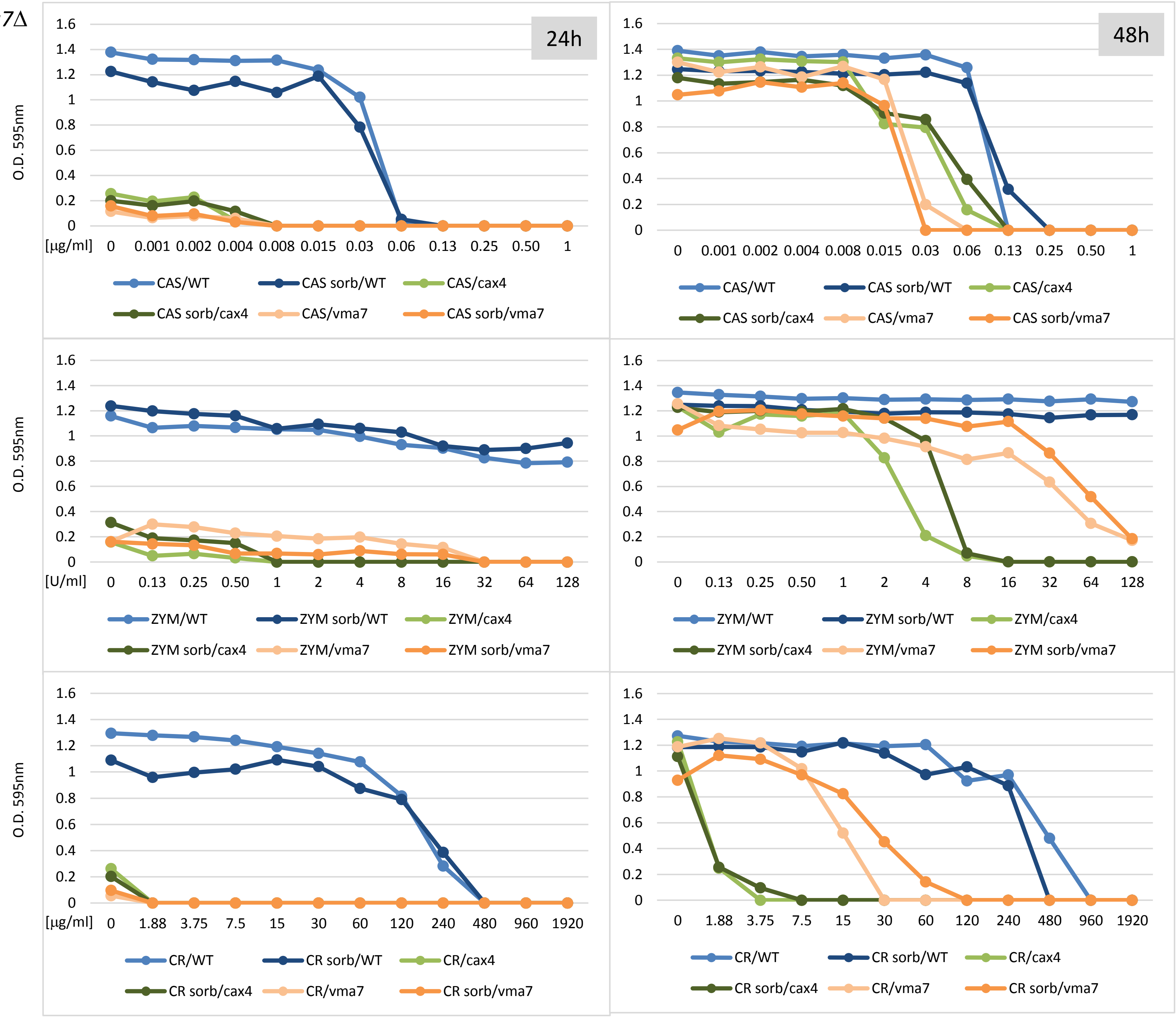

*vph2*Δ and *vma5*Δ

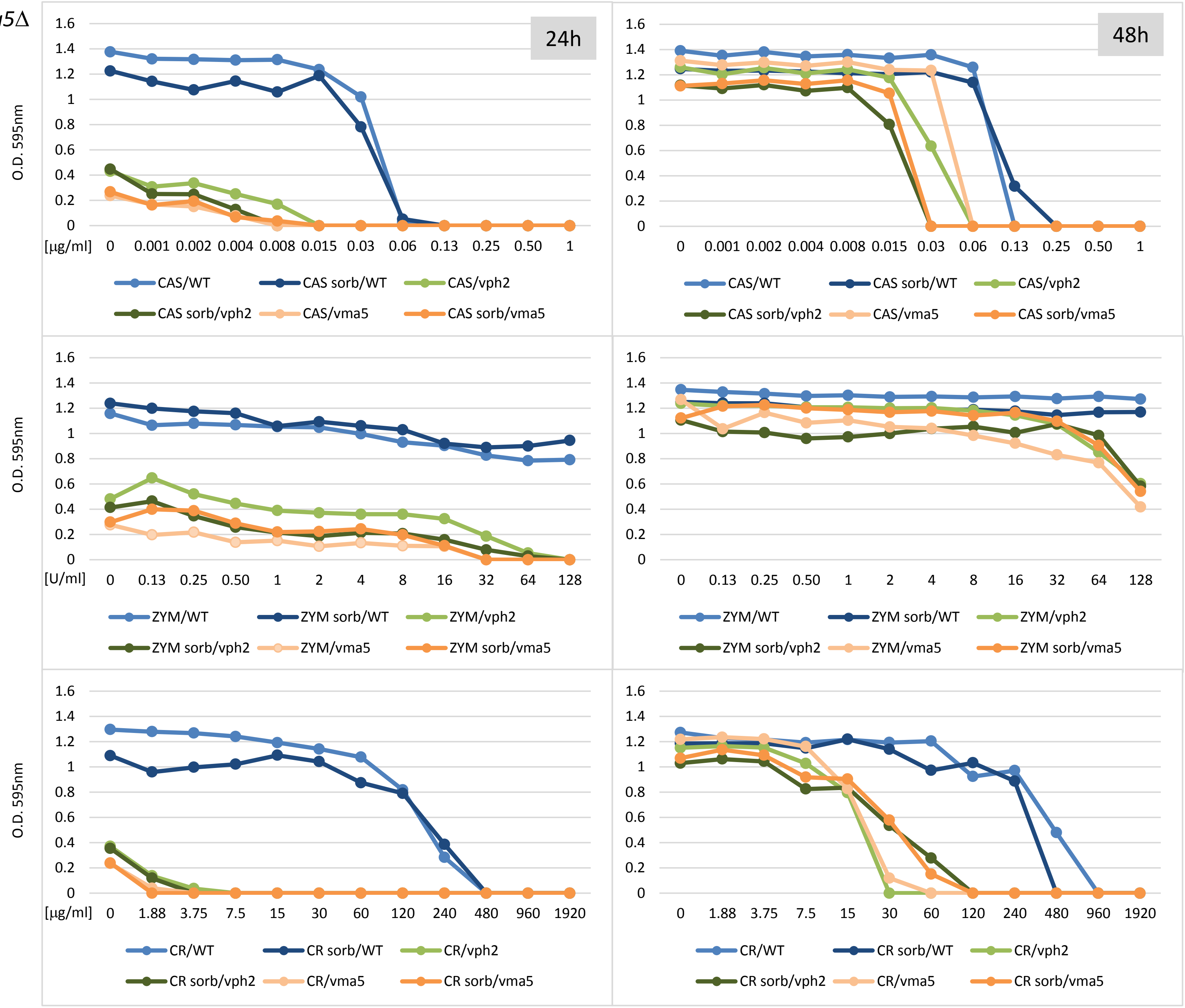

*fps1*Δ and *def1*Δ

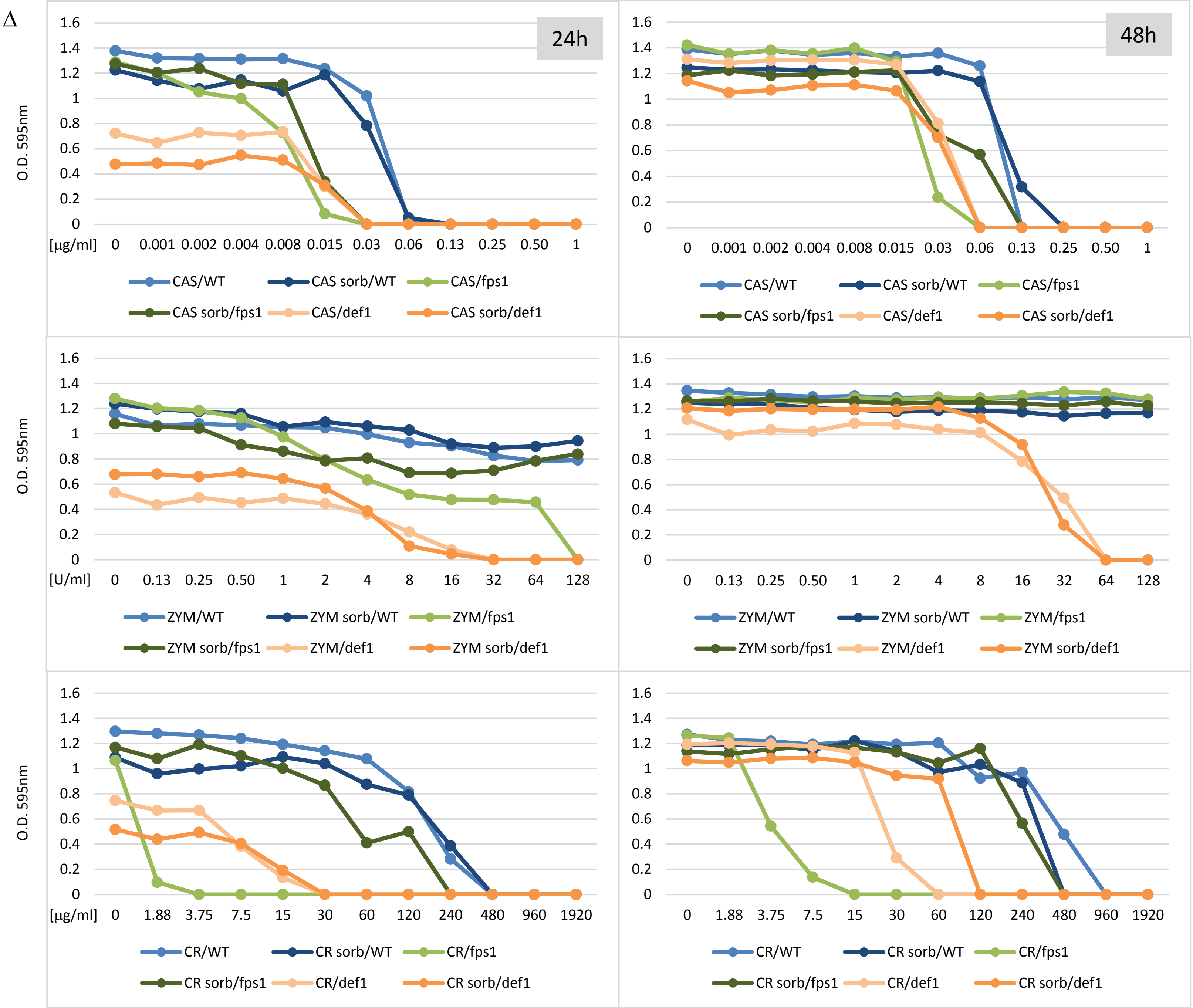

*scp160*Δ and *las21*Δ

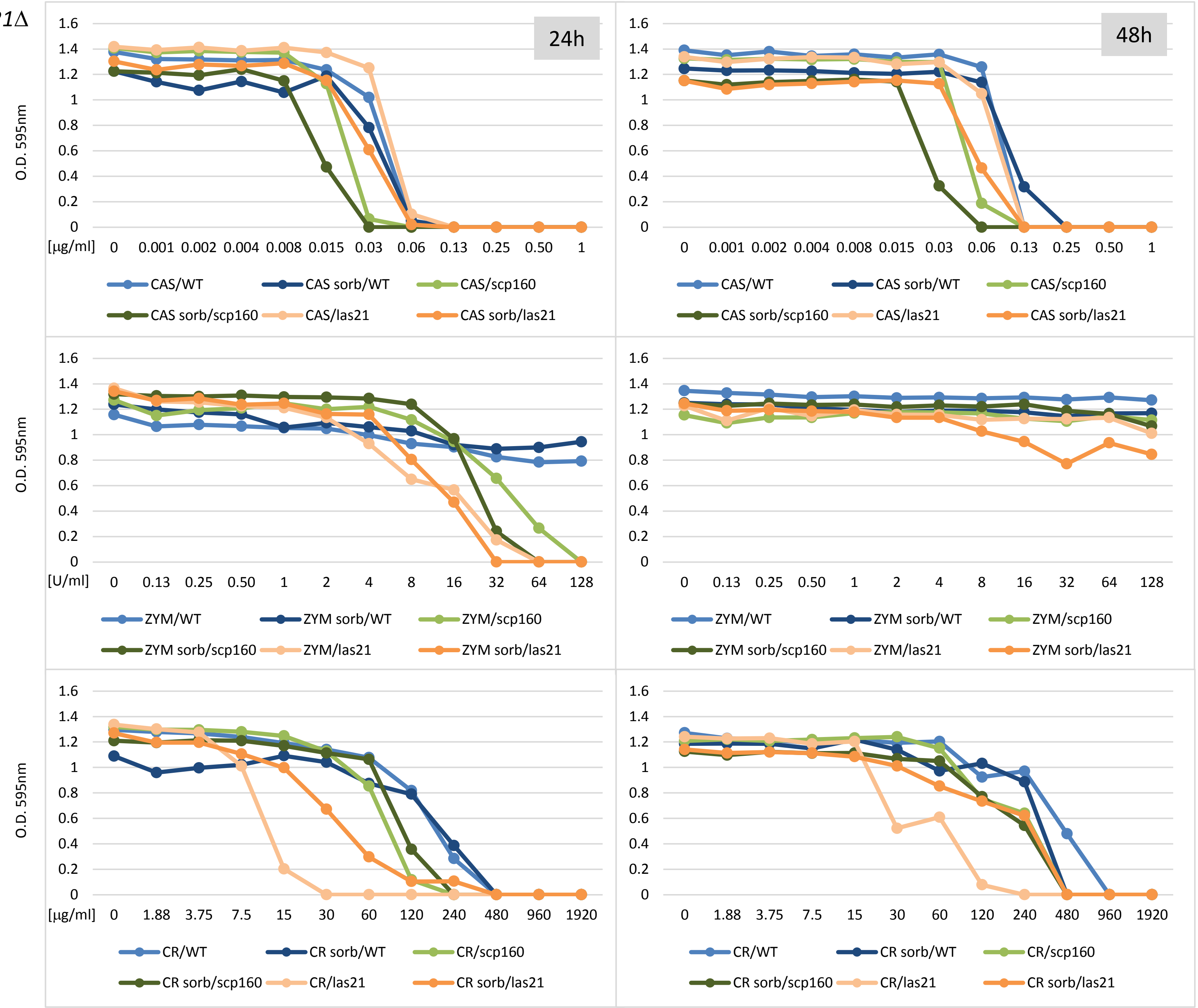

*bck1*Δ and *asf1*Δ

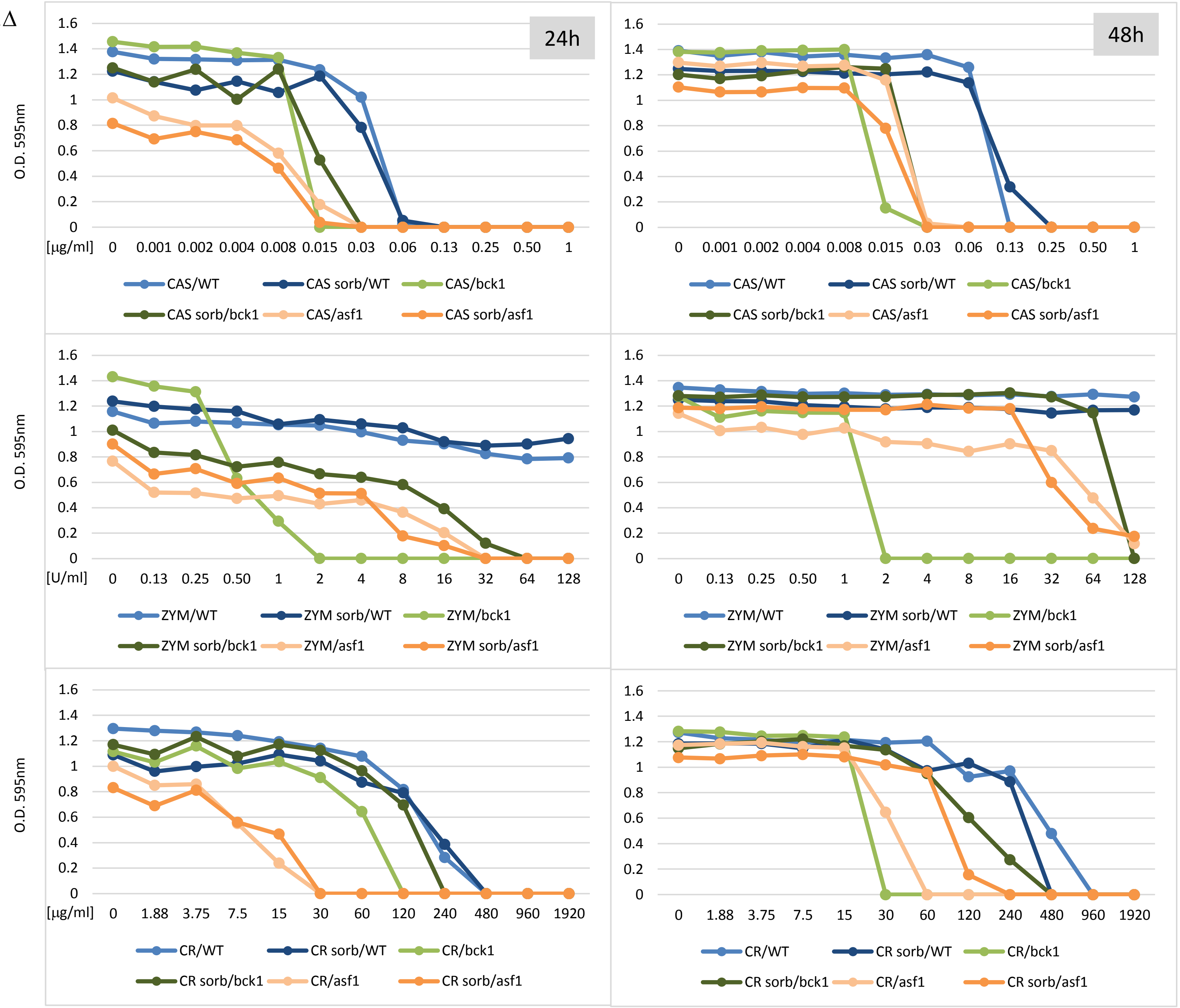

*snf1*Δ and *ies6*Δ

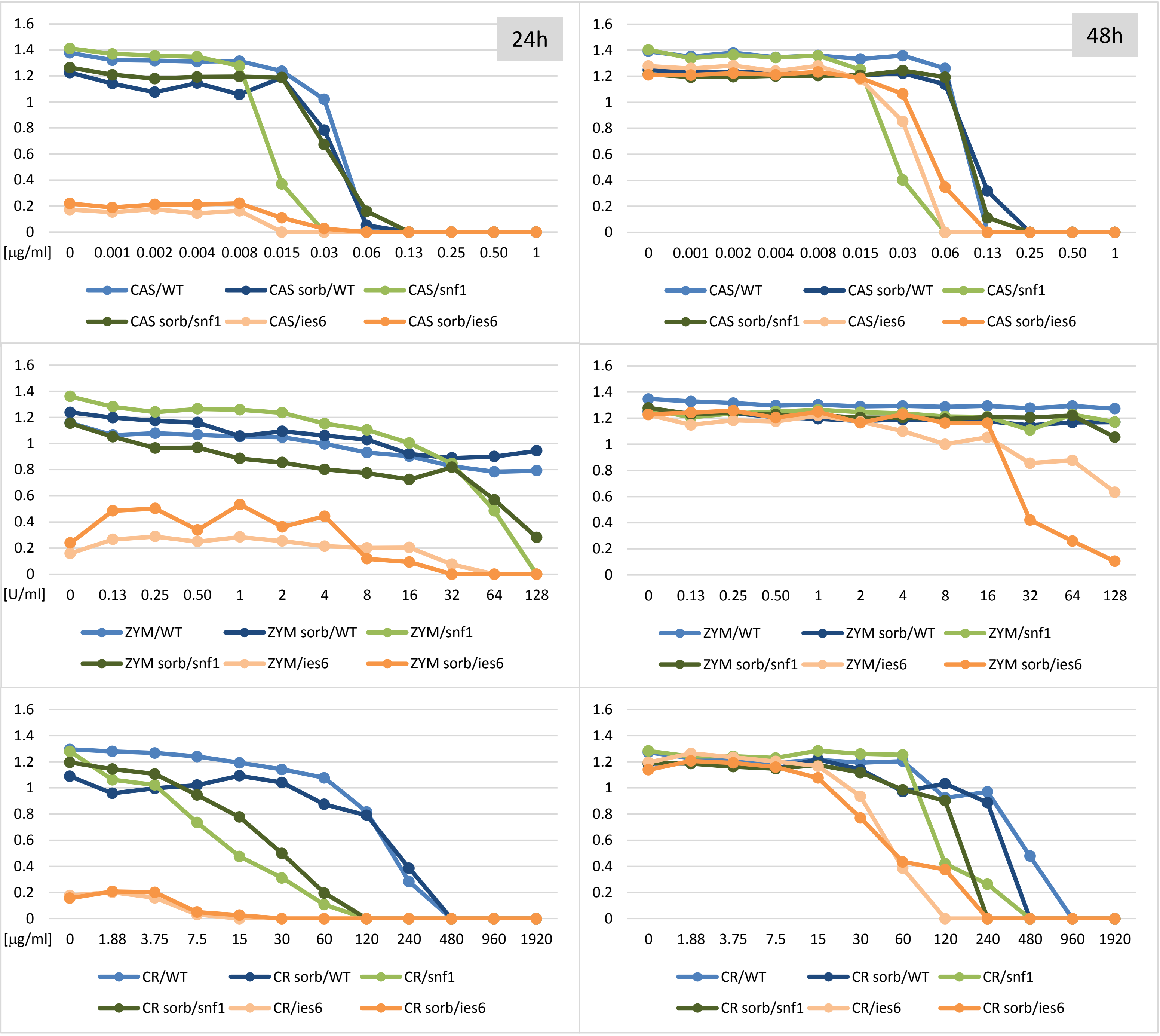

*bud32*Δ and *ypt6*Δ

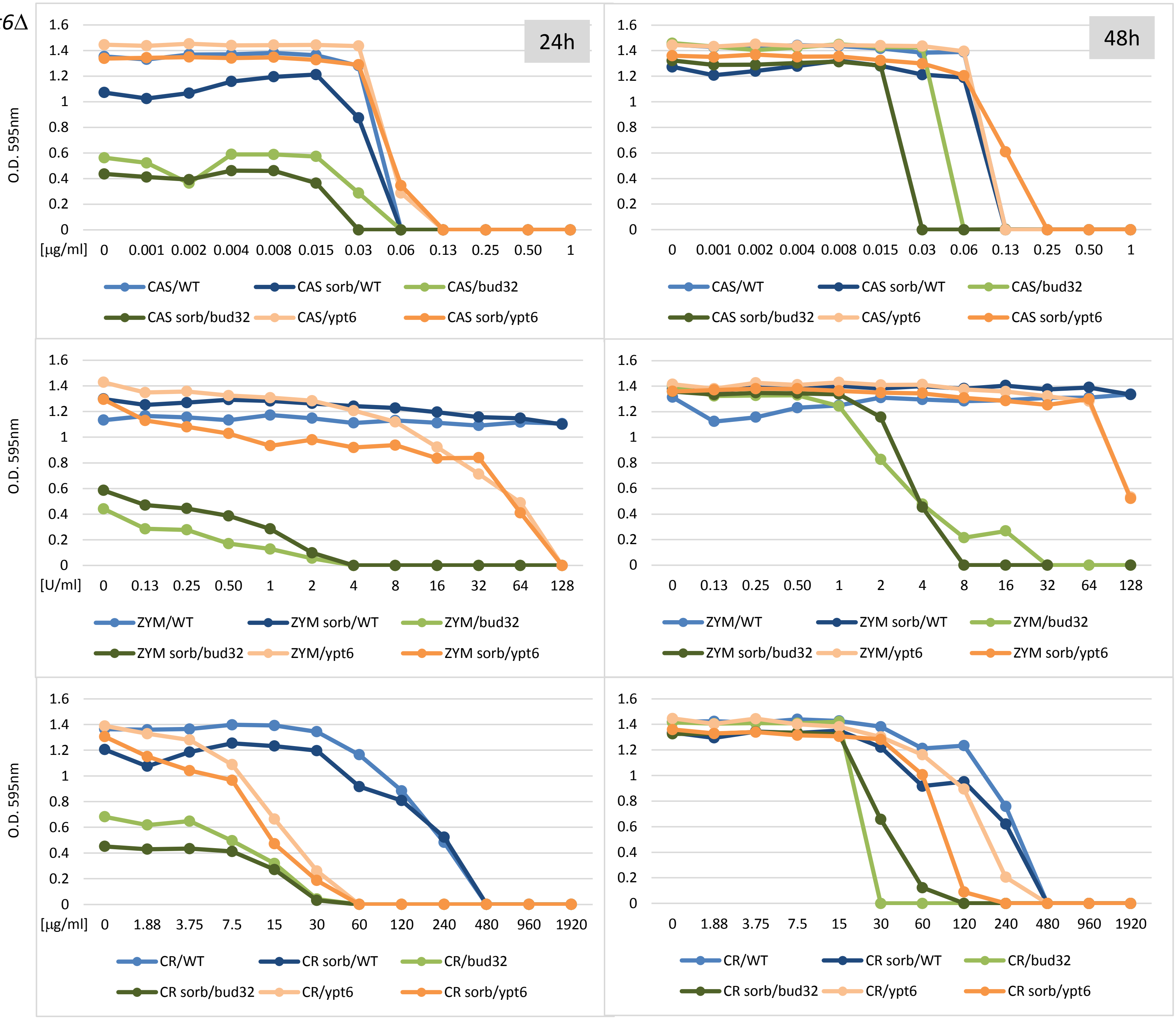

*mnn10*Δ and *akr1*Δ

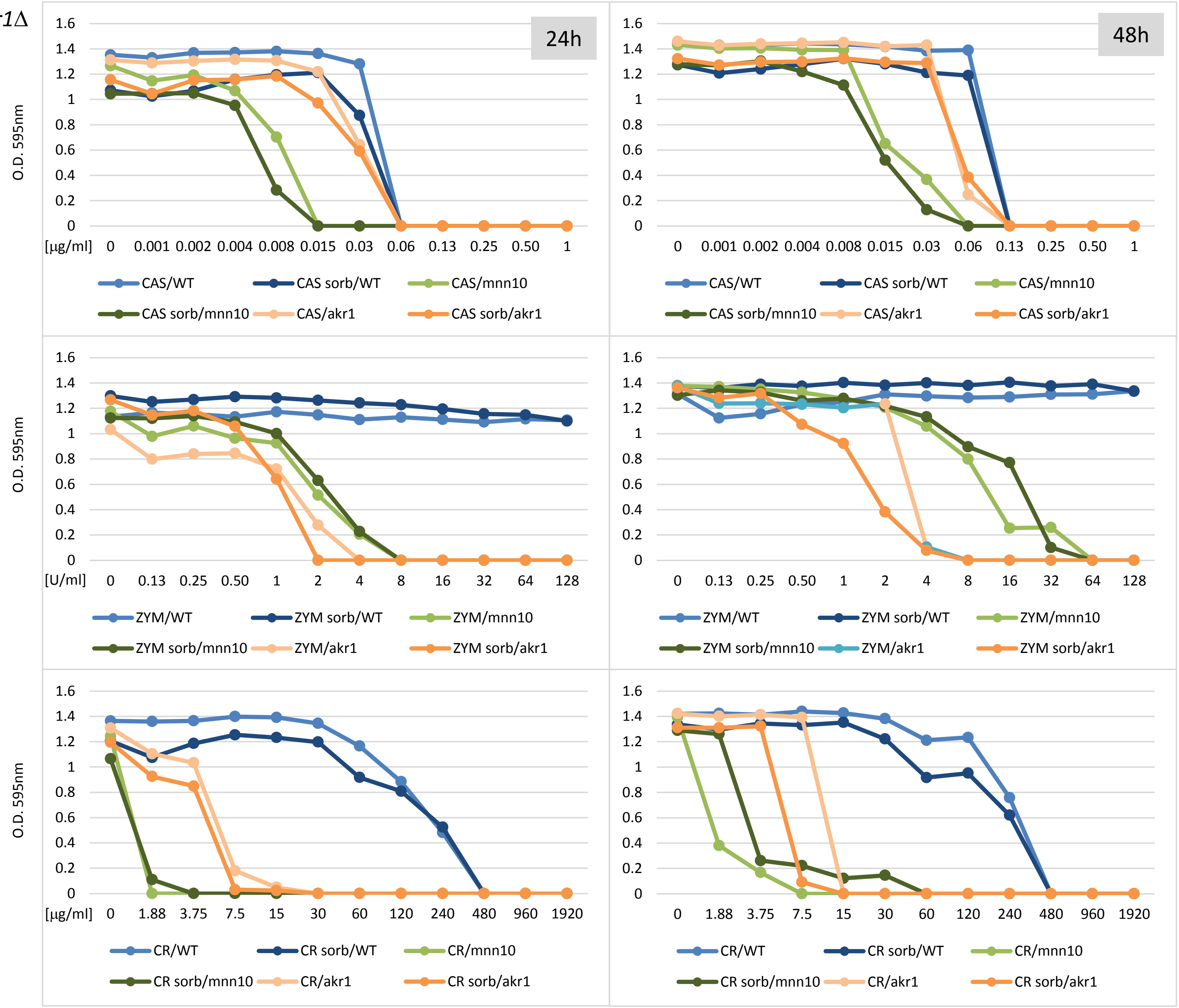

*pct1*Δ and *cdc40*Δ

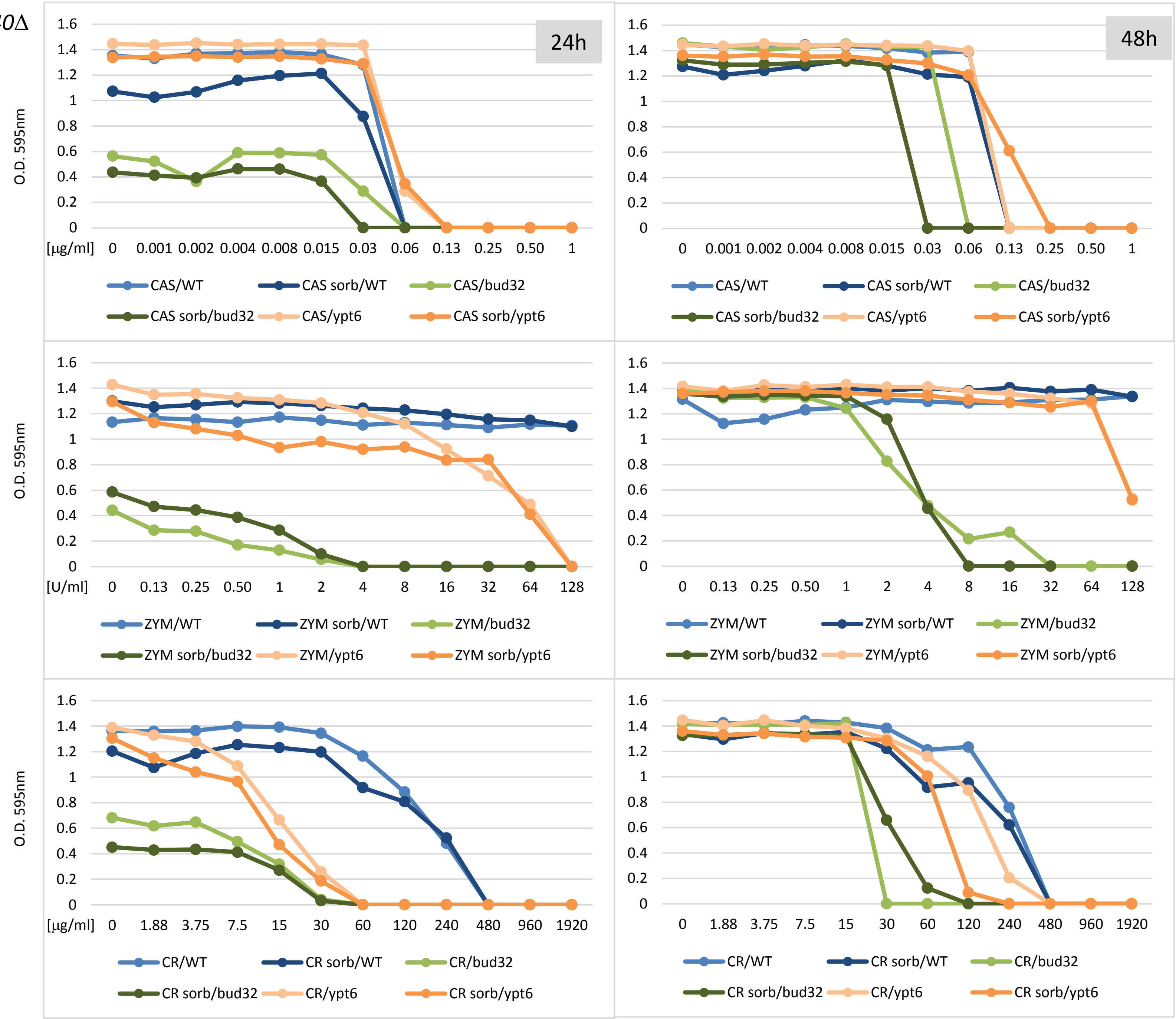

*she4*Δ and *erg6*Δ

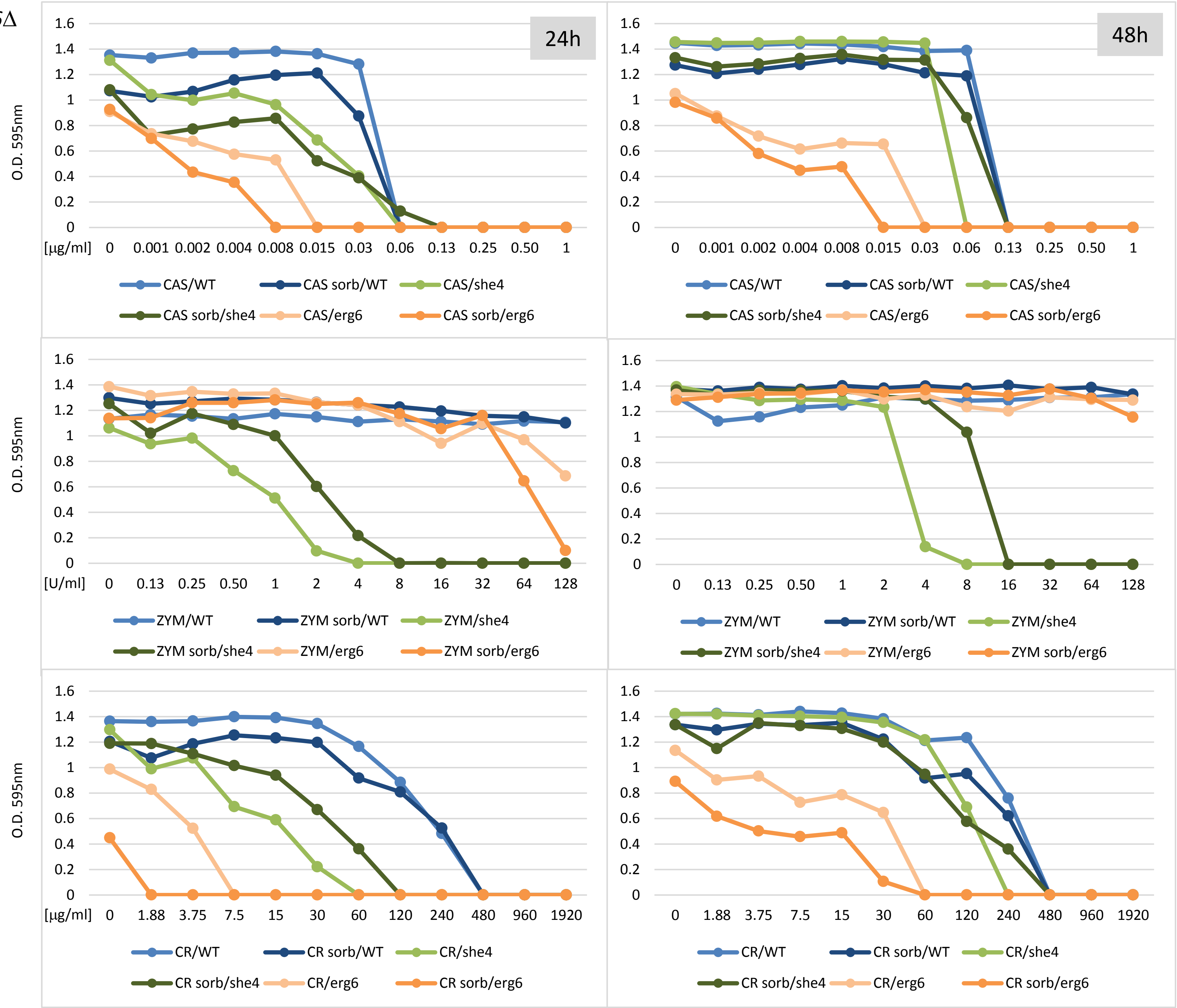

*gas1*Δ and *end3*Δ

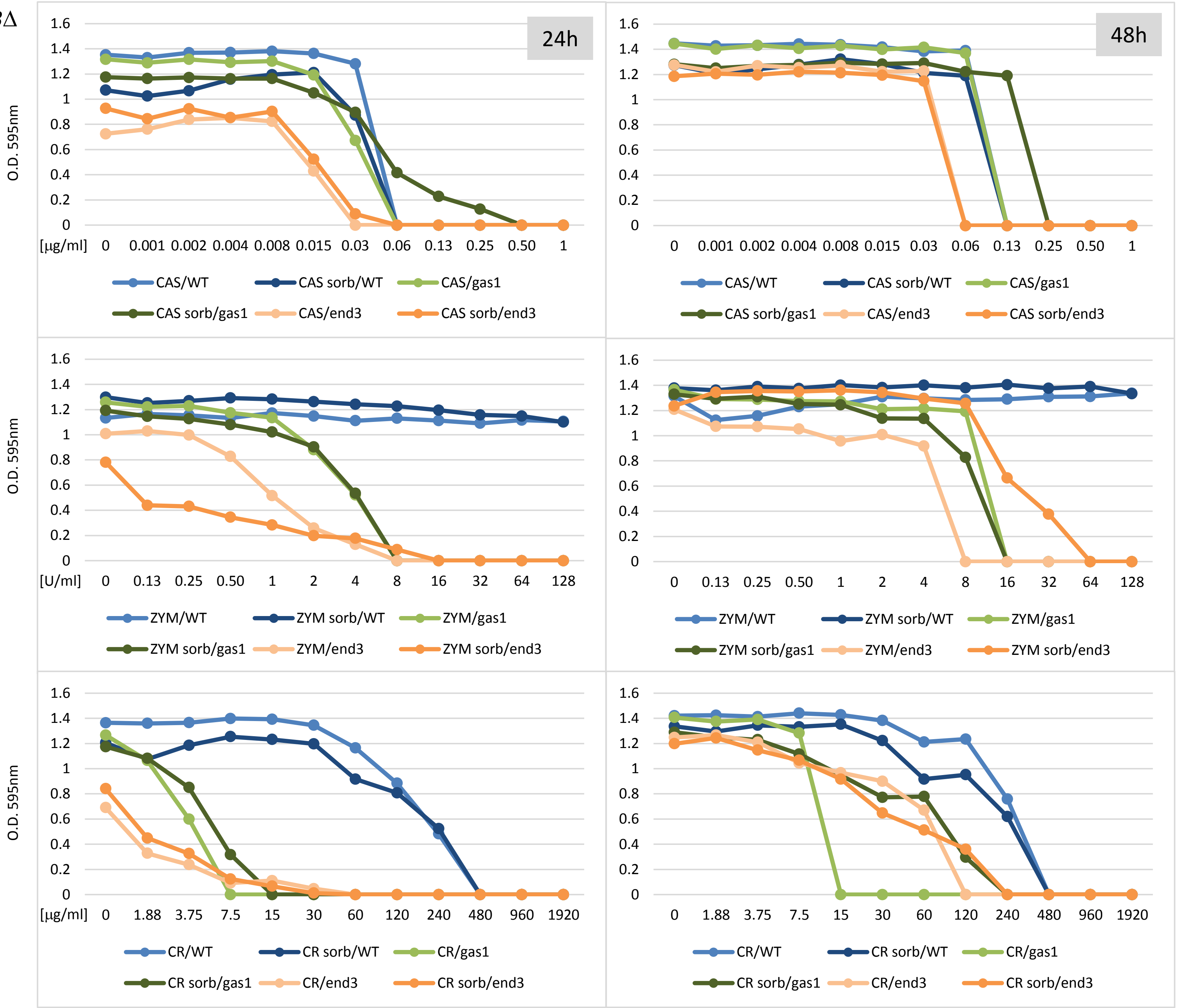

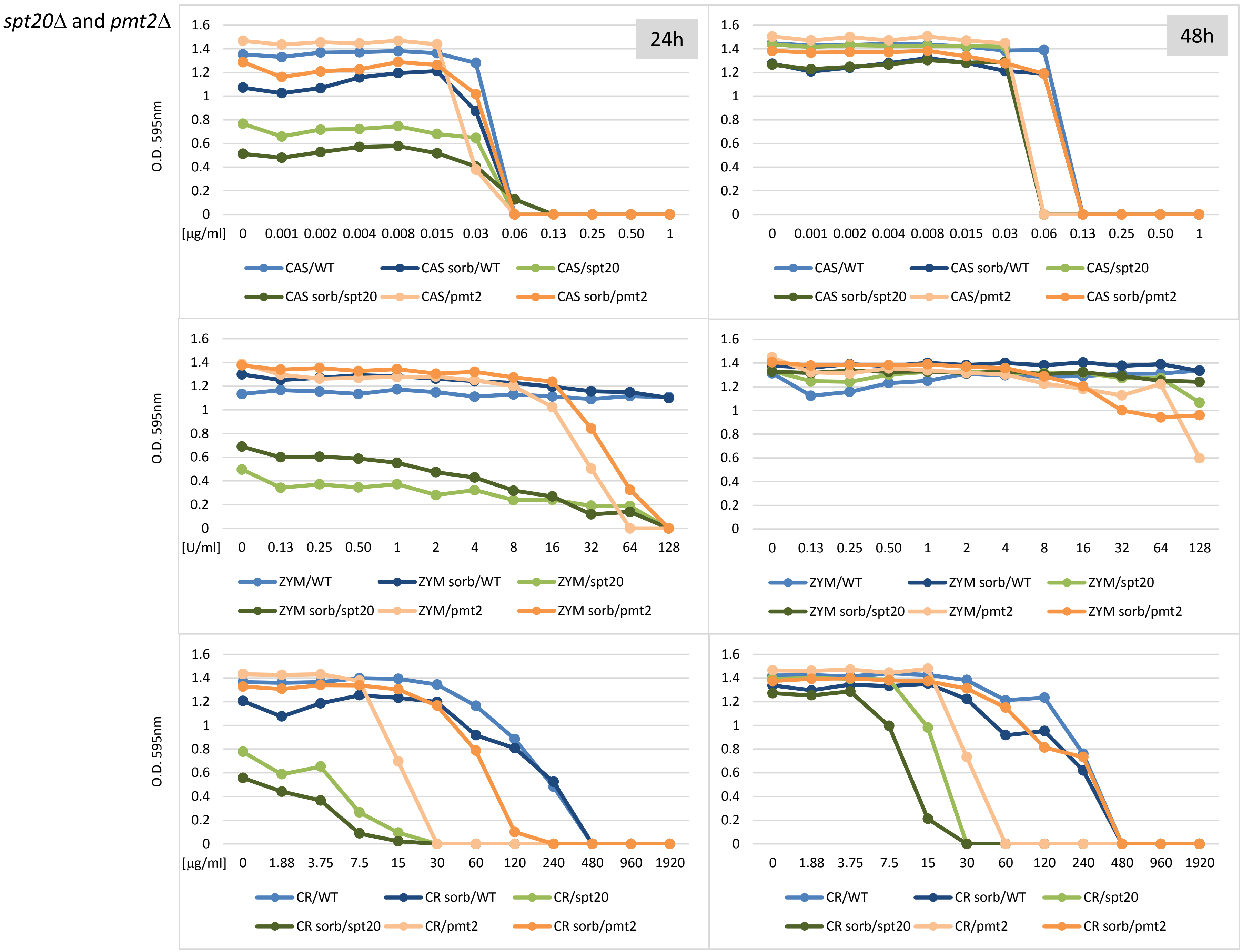
spt20Δ and pmt2Δ
24h
48h
O.D. 595nm
1.6
1.4
1.2
1
0.8
0.6
0.4
0.2
0
[µg/ml]
0 0.001 0.002 0.004 0.008 0.015 0.03 0.06 0.13 0.25 0.50 1
CAS/WT
CAS sorb/WT
CAS/spt20
CAS sorb/spt20
CAS/pmt2
CAS sorb/pmt2
[U/ml]
0 0.13 0.25 0.50 1 2 4 8 16 32 64 128
ZYM/WT
ZYM sorb/WT
ZYM/spt20
ZYM sorb/spt20
ZYM/pmt2
ZYM sorb/pmt2
[µg/ml]
0 1.88 3.75 7.5 15 30 60 120 240 480 960 1920
CR/WT
CR sorb/WT
CR/spt20
CR sorb/spt20
CR/pmt2
CR sorb/pmt2

*shp1*Δ and *rox3*Δ

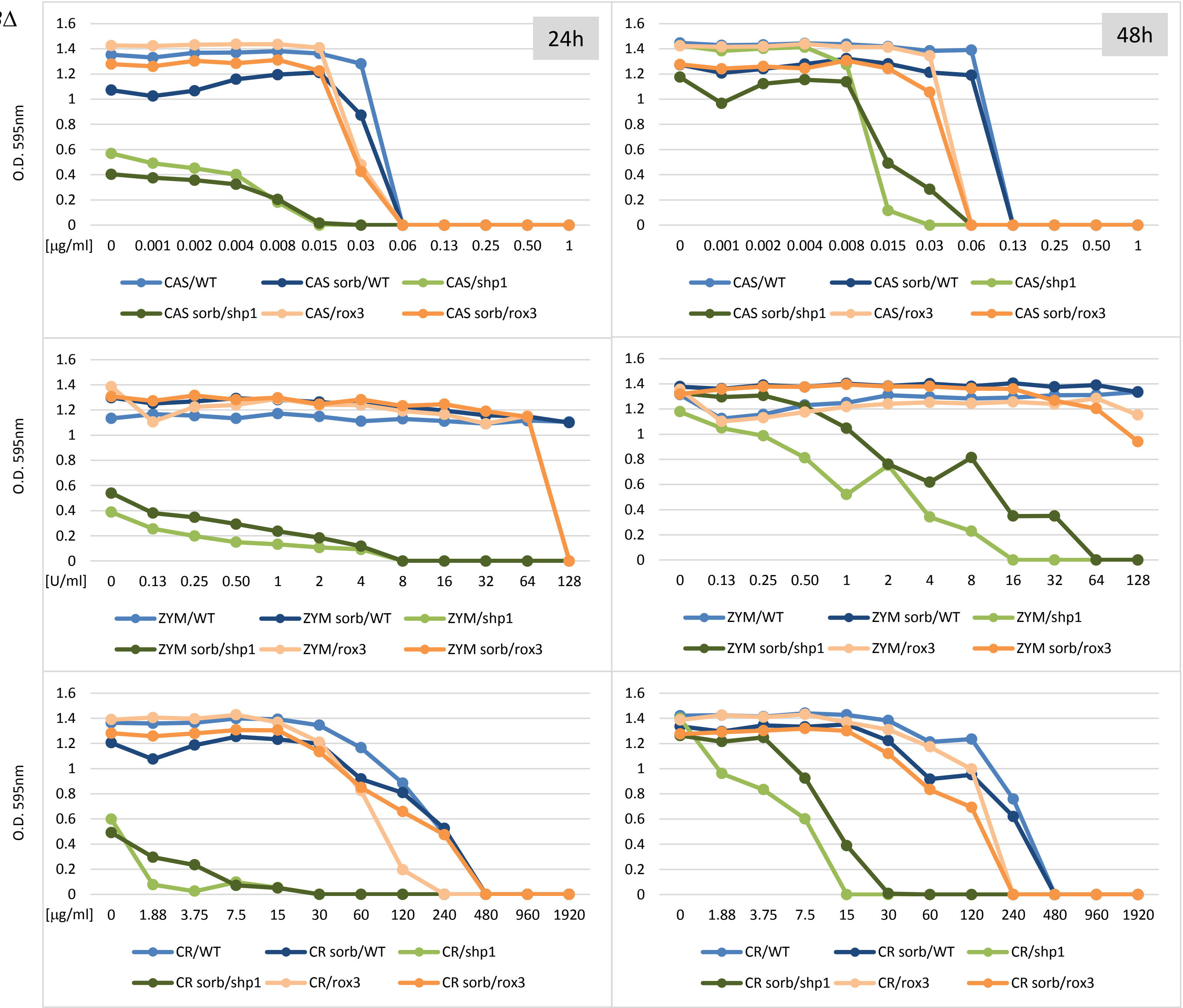

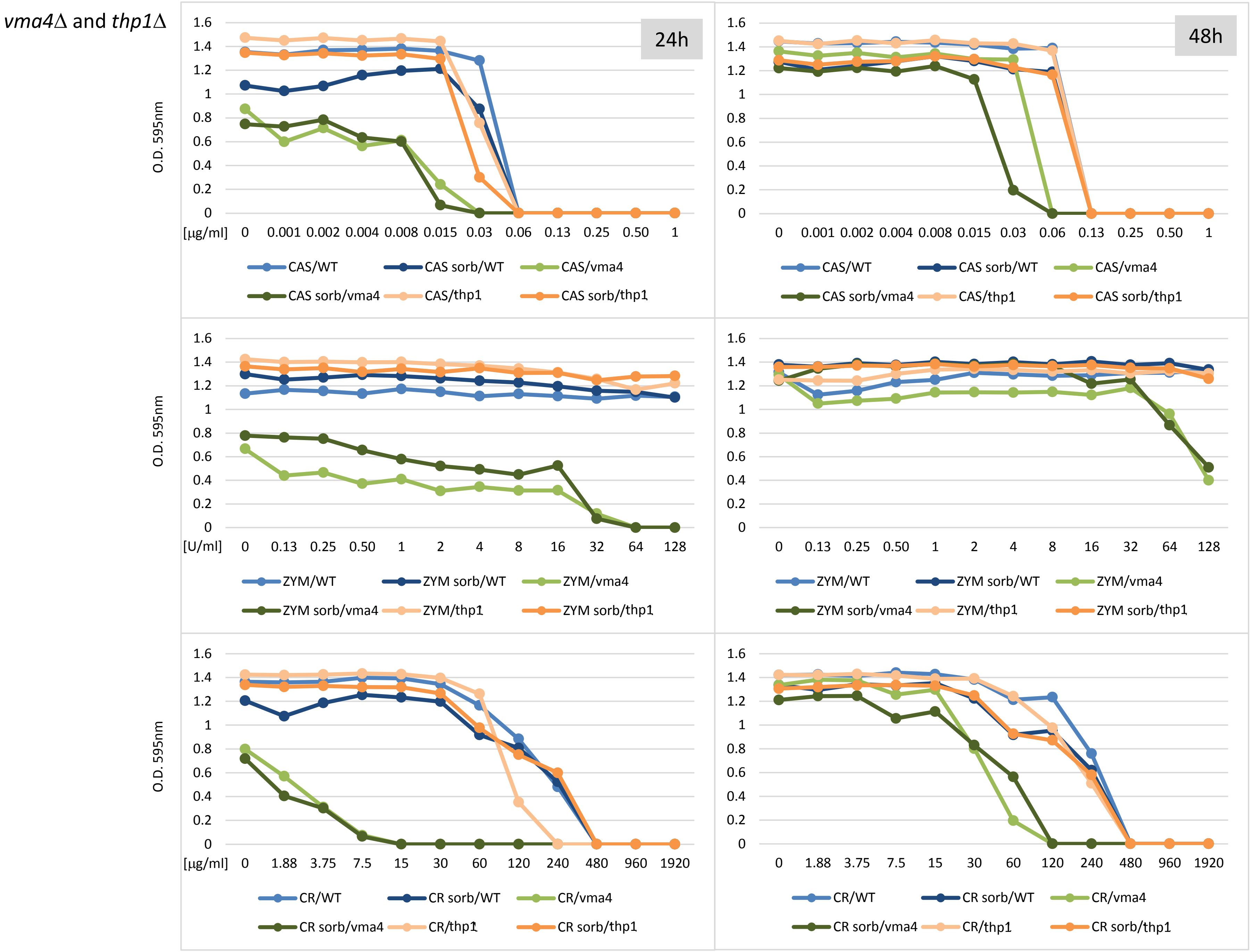
vma4Δ and thp1Δ
24h
48h
O.D. 595nm
1.6
1.4
1.2
1
0.8
0.6
0.4
0.2
0
[µg/ml]
0 0.001 0.002 0.004 0.008 0.015 0.03 0.06 0.13 0.25 0.50 1
CAS/WT
CAS sorb/WT
CAS/vma4
CAS sorb/vma4
CAS/thp1
CAS sorb/thp1
[U/ml]
0 0.13 0.25 0.50 1 2 4 8 16 32 64 128
ZYM/WT
ZYM sorb/WT
ZYM/vma4
ZYM sorb/vma4
ZYM/thp1
ZYM sorb/thp1
0 1.88 3.75 7.5 15 30 60 120 240 480 960 1920
CR/WT
CR sorb/WT
CR/vma4
CR sorb/vma4
CR/thp1
CR sorb/thp1

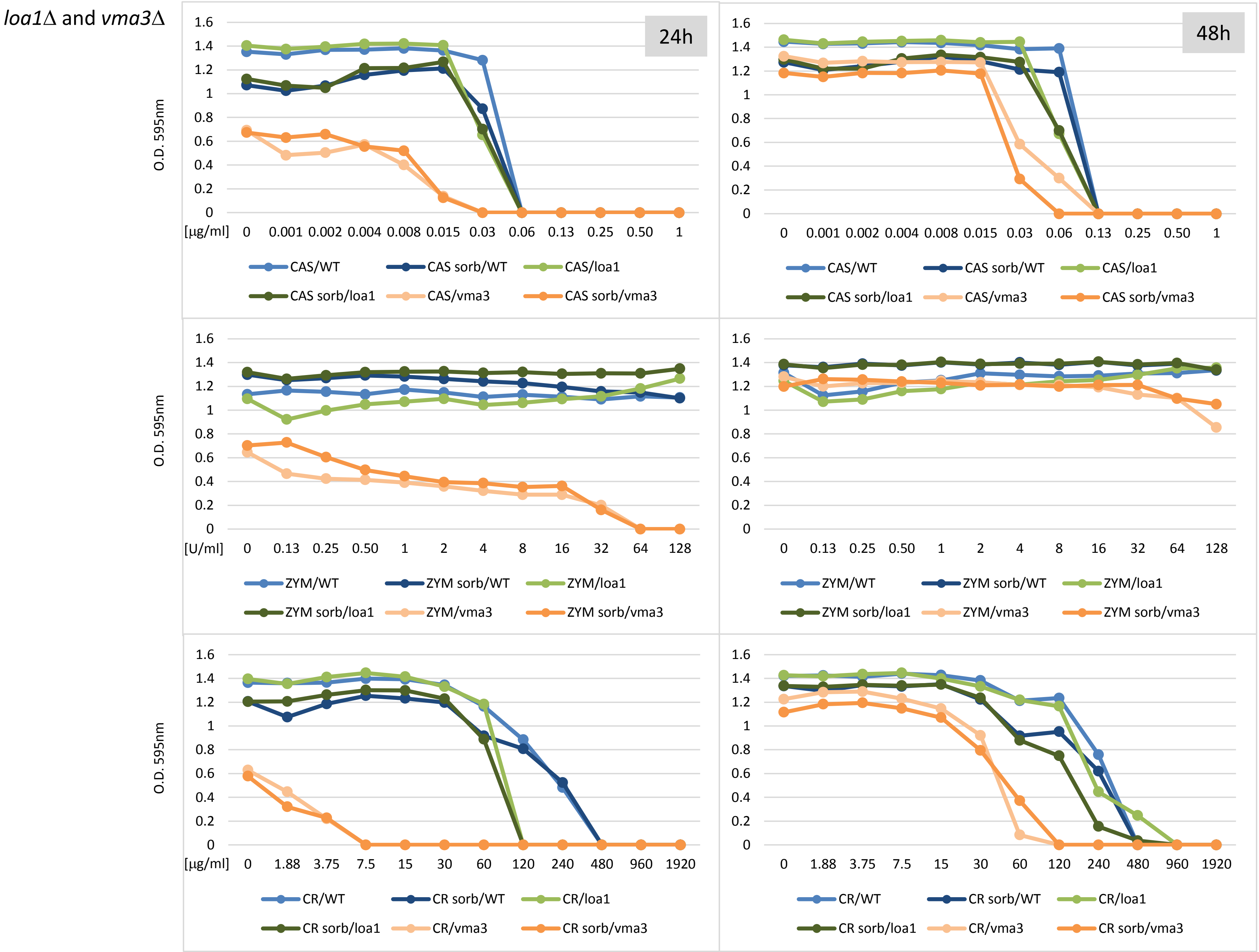
loa1Δ and vma3Δ
24h
48h
O.D. 595nm
[µg/ml]
0 0.001 0.002 0.004 0.008 0.015 0.03 0.06 0.13 0.25 0.50 1
CAS/WT
CAS sorb/WT
CAS/loa1
CAS sorb/loa1
CAS/vma3
CAS sorb/vma3
[U/ml]
0 0.13 0.25 0.50 1 2 4 8 16 32 64 128
ZYM/WT
ZYM sorb/WT
ZYM/loa1
ZYM sorb/loa1
ZYM/vma3
ZYM sorb/vma3
0 1.88 3.75 7.5 15 30 60 120 240 480 960 1920
CR/WT
CR sorb/WT
CR/loa1
CR sorb/loa1
CR/vma3
CR sorb/vma3
